# MMP^3^C v2: a network-based framework decoding metabolic plasticity in rheumatoid arthritis, enabling accurate diagnosis and uncovering cell-type-specific metabolic rewiring

**DOI:** 10.1093/bib/bbag307

**Published:** 2026-06-14

**Authors:** Xingyu Chen, Zihan Wang, Min Deng, Jianxiang Huang, Naishu Zhang, Zheng Wu, Zelin Yi, Sangyu Li, Jiayue Qiu, Kit-Leong Cheong, Xin Chen, Chen Huang

**Affiliations:** Dr. Neher’s Biophysics Laboratory for Innovative Drug Discovery, State Key Laboratory of Mechanism and Quality of Chinese Medicine & Faculty of Chinese Medicine, Macau University of Science and Technology, Avenida Wai Long, Taipa, Macau SAR 999078, China; Dr. Neher’s Biophysics Laboratory for Innovative Drug Discovery, State Key Laboratory of Mechanism and Quality of Chinese Medicine & Faculty of Chinese Medicine, Macau University of Science and Technology, Avenida Wai Long, Taipa, Macau SAR 999078, China; CRDA, Faculty of Health Sciences, University of Macau, Taipa, Macau SAR 999078, China; Dr. Neher’s Biophysics Laboratory for Innovative Drug Discovery, State Key Laboratory of Mechanism and Quality of Chinese Medicine & Faculty of Chinese Medicine, Macau University of Science and Technology, Avenida Wai Long, Taipa, Macau SAR 999078, China; Dr. Neher’s Biophysics Laboratory for Innovative Drug Discovery, State Key Laboratory of Mechanism and Quality of Chinese Medicine & Faculty of Chinese Medicine, Macau University of Science and Technology, Avenida Wai Long, Taipa, Macau SAR 999078, China; Dr. Neher’s Biophysics Laboratory for Innovative Drug Discovery, State Key Laboratory of Mechanism and Quality of Chinese Medicine & Faculty of Chinese Medicine, Macau University of Science and Technology, Avenida Wai Long, Taipa, Macau SAR 999078, China; Dr. Neher’s Biophysics Laboratory for Innovative Drug Discovery, State Key Laboratory of Mechanism and Quality of Chinese Medicine & Faculty of Chinese Medicine, Macau University of Science and Technology, Avenida Wai Long, Taipa, Macau SAR 999078, China; Dr. Neher’s Biophysics Laboratory for Innovative Drug Discovery, State Key Laboratory of Mechanism and Quality of Chinese Medicine & Faculty of Chinese Medicine, Macau University of Science and Technology, Avenida Wai Long, Taipa, Macau SAR 999078, China; Dr. Neher’s Biophysics Laboratory for Innovative Drug Discovery, State Key Laboratory of Mechanism and Quality of Chinese Medicine & Faculty of Chinese Medicine, Macau University of Science and Technology, Avenida Wai Long, Taipa, Macau SAR 999078, China; College of Food Science and Technology, Guangdong Provincial Key Laboratory of Aquatic Product Processing and Safety, Guangdong Province Engineering Laboratory for Marine Biological Products, Guangdong Provincial Engineering Technology Research Center of Seafood, Guangdong Provincial Engineering Technology Research Center of Prefabricated Seafood Processing and Quality Control, Guangdong Ocean University, No. 1 Haida Road, Mazhang District, Zhanjiang City, Guangdong Province 524088, China; School of Automation, Guangdong University of Technology, No. 100 Waihuan Xi Road, Guangzhou Higher Education Mega Center, Panyu District, Guangzhou, Guangdong Province 510006, China; Dr. Neher’s Biophysics Laboratory for Innovative Drug Discovery, State Key Laboratory of Mechanism and Quality of Chinese Medicine & Faculty of Chinese Medicine, Macau University of Science and Technology, Avenida Wai Long, Taipa, Macau SAR 999078, China

**Keywords:** metabolic plasticity, rheumatoid arthritis, biomarker, scRNA-seq, diagnosis

## Abstract

Metabolic plasticity, the ability of cells to dynamically adapt their metabolic pathways in response to changing environments, is a hallmark of rheumatoid arthritis (RA) pathogenesis and plays a critical role in immune dysfunction. However, scalable methods to quantify inter-pathway crosstalk in RA remain lacking. To address this gap, we present MMP^3^C v2, an updated network-based framework that integrates gene expression with protein–protein interaction network topology to compute directed pairwise metabolic plasticity (PMP) scores. We applied MMP^3^C v2 to ~3400 bulk transcriptomes (RA, osteoarthritis, systemic lupus erythematosus, and healthy controls) and ~228 000 single-cell transcriptomics from blood and synovium to profile RA-associated PMP alterations and develop diagnostic classifiers. We found that a single PMP-derived signature demonstrated strong predictive capability for diagnosis. Then, we developed a feature selection pipeline and combined it with 110 machine learning model combinations, by which we established the optimal ensemble classifier (stepwise forward selection + ridge regression), achieving robust and generalized performance (mean area under the curve (AUC) = 0.935; mean F1 score = 0.915) across 12 independent validation cohorts, outperforming seven previously published models. Single-cell analysis revealed cell-type-specific PMP remodeling: a Warburg-like shift in synovial macrophages (↑glycolysis, ↑pentose phosphate pathway, ↓oxidative phosphorylation). Cell–cell communication analysis highlighted FN1-centered signaling linked to glucose metabolic remodeling in myofibroblasts. Collectively, MMP^3^C v2 establishes metabolic pathway crosstalk as a core diagnostic feature of RA, enabling interpretable and cross-platform diagnostic modeling and the identification of cell-type-specific PMP patterns. The open-source R package mmp3c supports reproducible analysis and broad application.

## Graphical abstract

**Graphical Abstract:**
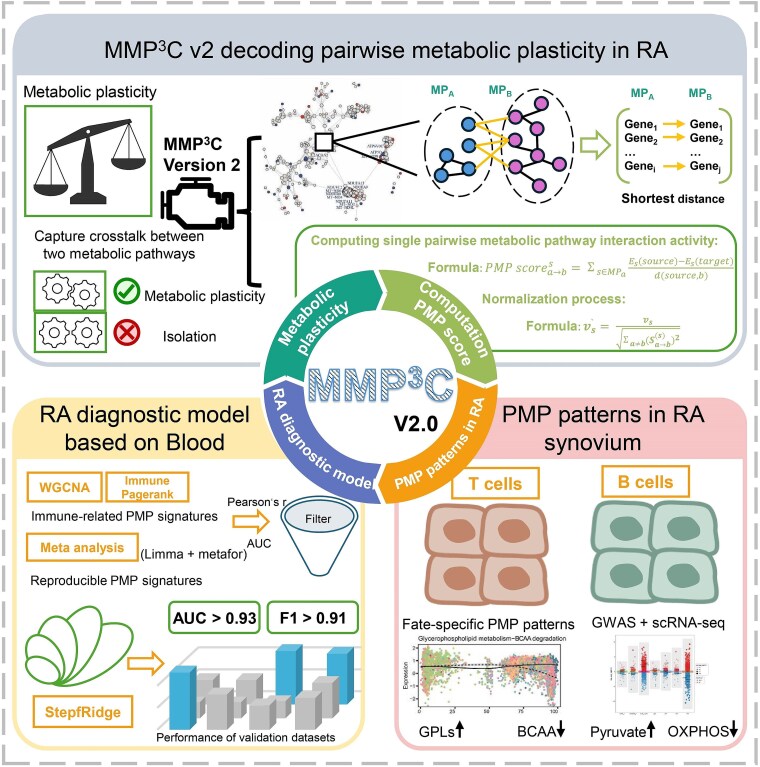

## Introduction

A century ago, Warburg observed that tumors preferentially utilize glycolysis rather than oxidative phosphorylation (OXPHOS) for glucose metabolism even under oxygen-rich conditions [1]. Since then, increasing evidence has shown that alterations in cellular metabolism can profoundly reshape immune cell composition and function [2–4], and that analogous metabolic shifts occur in immune-mediated diseases such as rheumatoid arthritis (RA). RA is a systemic autoimmune disease characterized by chronic synovitis. Within joints, the synovial microenvironment imposes severe environmental stresses on resident and infiltrating cells, notably profound hypoxia, nutrient deprivation, oxidative stress, and persistent inflammation—conditions that promote glycolytic metabolism via HIF-1α and related signaling programs, similar to those observed in cancer [5]. Despite clinical advances, early and specific diagnosis of RA remains challenging: commonly used serological markers (e.g. RF, anti-CCP) and many proposed molecular biomarkers often lack sensitivity, specificity, or reproducibility across cohorts and platforms [6, 7]. Accordingly, there is an urgent need to develop robust, translatable biomarkers that can identify patients within the therapeutic “window of opportunity” [8], enabling timely intervention and improved long-term outcomes [9].

In RA, inflammatory and tissue-derived cues can rapidly shift immune and stromal cells from a quiescent to an activated metabolic state, reflecting the disease’s dynamic, context-dependent metabolic plasticity. This shift is partly mediated through PI3K–Akt and mTOR signaling, which promote glycolytic reprogramming and support pro-inflammatory effector functions [10]. Importantly, metabolic plasticity in RA is not restricted to the up- or down-regulation of individual metabolic pathways; rather, it involves dynamic redistribution of metabolic activity across interconnected pathways—for example, between glycolysis, the pentose phosphate pathway (PPP), and OXPHOS [11, 12]. Different cell types exhibit distinct metabolic signatures (e.g. glycolysis-dominant M1-like macrophages versus OXPHOS-reliant Tregs), thereby reshaping immune cell phenotypes and functions. Metabolic heterogeneity among immune and stromal cells has been documented in synovium and peripheral blood, and this cell-level metabolic reprogramming is detectable systemically in RA—for example, altered levels of glycerophosphocholine and trans-cinnamic acid have been reported in patient plasma [13, 14]. Despite these advances, most experimental and computational studies remain restricted to single-pathway or low-throughput analyses and, therefore, underappreciate the pairwise pathway crosstalk and the influence of network topology on metabolic remodeling—gaps that motivate network-aware approaches such as pairwise metabolic plasticity (PMP).

**Figure 1:**
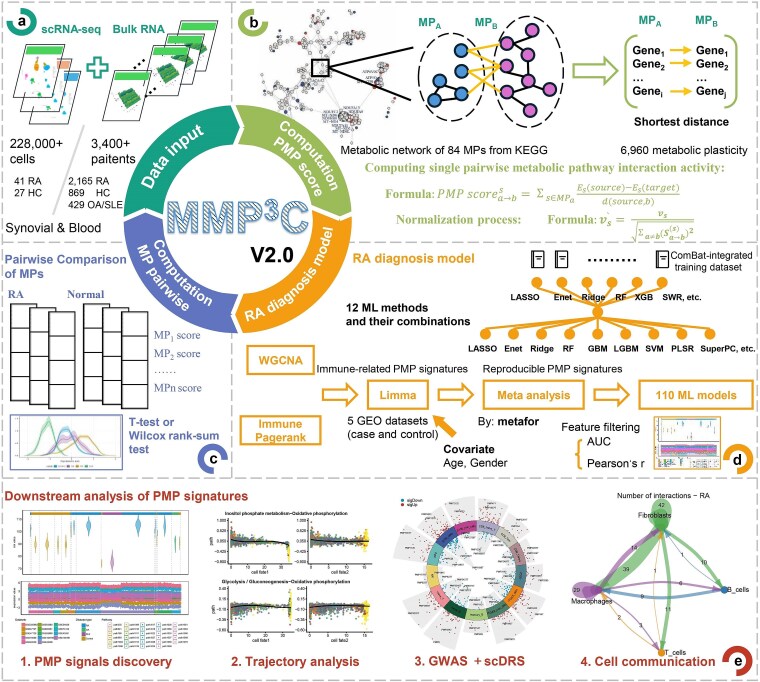
Architecture of the MMP^3^C v2 framework for unveiling metabolic pathway interaction plasticity in RA through pairwise comparisons. (a) Collection of RA-related transcriptomic datasets, including bulk RNA-seq and scRNA-seq datasets. (b) Construction of directed PMP interaction scores by integrating gene expression with PPI network topology. (c) Comparison of metabolic plasticity patterns across different disease states. (d) Development of an RA diagnostic model based on PMP signatures. (e) Application of PMP signatures to various downstream analyses.

We present MMP^3^C v2, an *in-silico* framework that quantifies PMP by integrating gene expression with protein–protein interaction (PPI) network topology (see workflow in Fig. 1). Our core hypothesis is that metabolically interacting pathways tend to occupy nearby regions in the PPI network, and that combining network proximity with differential expression reveals putative inter-pathway influence [15]. MMP^3^C v2 identifies RA-specific PMP patterns with higher accuracy compared to healthy controls and disease controls, including osteoarthritis (OA) and systemic lupus erythematosus (SLE). Importantly, PMP-derived signatures can be translated into cross-platform diagnostic models [mean area under the curve (AUC) 0.906 for single-indicator; ensemble mean AUC 0.935 across 12 cohorts] that outperform existing single-gene biomarkers and diagnostic models, offering a translational route towards earlier, biology-informed diagnosis of RA. At single-cell resolution, the framework characterizes cellular metabolic plasticity states across disease types and stages, providing insights into RA progression.

## Results

### The MMP^3^C v2 framework for modeling RA pairwise metabolic plasticity

The original MMP^3^C framework [16] quantified metabolic plasticity by integrating gene expression with intra-pathway network topology, treating each pathway in isolation. However, this original approach lacked contextual biological constraints linking pathways and, therefore, was prone to false-positive signals. To overcome this, MMP^3^C v2 introduces a critical new dimension: the inter-pathway network distance within the PPI network, which serves as a structural proxy for their potential functional interplay (Fig. 1; Methods). By jointly modeling inter-pathway network proximity alongside coordinated gene expression shifts, MMP3C v2 provides a more robust, contextually integrated view of pairwise metabolic plasticity in RA.

#### A dual-layered framework for quantifying pairwise metabolic plasticity

The framework quantifies the functional proximity between two metabolic pathways (MPs) using a high-confidence human PPI network knowledge curated from experimentally validated datasets [17]. It then captures the metabolic shifts across different disease states by comparing individualized PMP interaction scores (Fig. 1b). PMP interaction scores represent a computationally derived measure of how genes within one pathway (MP_a_) interact with genes in another pathway (MP_b_), incorporating both gene expression differences and the network proximity between genes (see Methods). Concretely, for each ordered MP pair (A and B) and for each gene g in the source MP_a_, we identified its closest connected gene h_g_ within the target pathway MP_b_ in terms of shortest-path distance in the PPI network [15, 17]. The shortest-path distance dist(g, hg) was used as a gene–pathway proximity distance reflecting how closely they are positioned in the PPI. To compute a sample-specific directional interaction score S(MP_a_ ➔ MP_b_), we integrated gene expression differences with these network distances. For a given sample, the score was calculated as the sum over all genes g in MP_a_ of the weighted divergence between gene g and its nearest gene h_g_ in the MP_b_ network: (expr(g)-expr(h_g_))/dist(g, h_g_). Here, the reciprocal of the distance (1/dist) serves as a weighting factor, prioritizing gene-MP pairs with closer network proximity [17] (Fig. 1b). The directional PMP score reflects asymmetric pathway perturbations within the network, rather than direct causality. For instance, a strong positive directional score from pathway A to pathway B indicates a stronger asymmetric network-based interaction from pathway A to pathway B than vice versa. The PMP interaction score provides a computationally tractable method for characterizing asymmetric pathway relationships within the metabolic network and may serve as a hypothesis-generating framework for further experimental validation.

Using 84 metabolic pathways within the Kyoto Encyclopedia of Genes and Genomes (KEGG) database (Table S1) [18], we derived a comprehensive PMP score matrix covering 6960 directed pathway pairs (excluding within-pathway pairs; Table S2). To ensure cross-sample comparability, raw PMP scores were normalized using cosine-based per-sample scaling (Fig. 1b; Methods). Differential PMP scores between the RA and control groups were assessed using two-sample t-tests with false discovery rate (FDR) correction (Fig. 1c). Finally, Shapley Additive Explanations (SHAP) analysis was applied to deconstruct the contribution of individual gene–gene interactions to RA–control differences in PMP scores, thereby identifying the genes most influential in disease classification (Supplementary Methods).

#### Framework validation and comparative advantage

**Figure 2:**
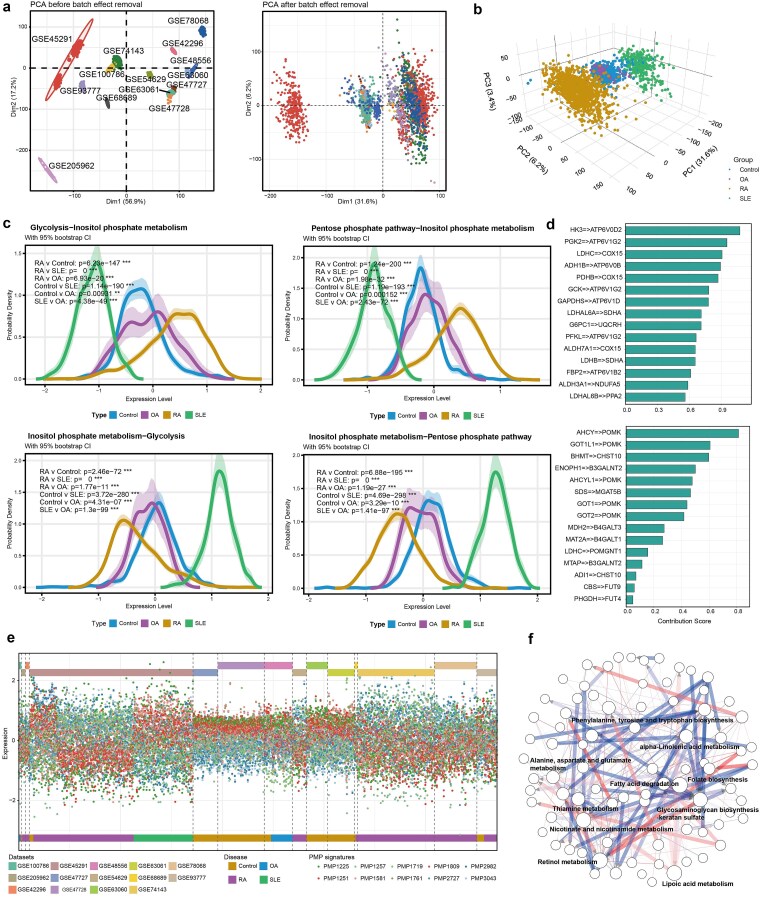
Pairwise cross-pathway metabolic plasticity characterized in multiple RA cohorts. (a) PCA analysis reveals the batch effect across multiple datasets from various technology platforms. (b) 3D PCA plot shows the difference among various disease states after batch correction. (c) The density distribution plots of bidirectional normalized PMP interactions across various disease states including RA, healthy controls, SLE, and OA. Representative pathway pairs are shown: Glycolysis → Inositol phosphate metabolism, Pentose phosphate pathway → Inositol phosphate metabolism. (d) Contribution analysis by MMP^3^C v2 for the pair of Glycolysis → Inositol phosphate metabolism (top) and the pair of Pentose phosphate pathway → Inositol phosphate metabolism (bottom). (e) Scatter plot of differential PMP signatures in the integrated cohorts across multiple disease states. (f) Network analysis revealed crucial metabolic pathway nodes in the RA-specific metabolic architecture.

We next compared MMP^3^C v2 to MMP^3^C v1 based on the ComBat-integrated dataset, consisting of RA patients (*n* = 1026), SLE (*n* = 292), OA (*n* = 112), and healthy controls (*n* = 683; Fig. 2a and b and cohort details in Table S3). MMP^3^C v2 demonstrated an improved capability to identify RA-associated metabolic plasticity patterns—including glucose, fatty acid, glutathione, and folate metabolism—compared with MMP^3^C v1 (Fig. S1A). We further benchmarked both frameworks against a canonical metabolic shift (Warburg effect: glycolysis versus OXPHOS) using pan-cancer transcriptomic data from The Cancer Genome Atlas Program (TCGA) database [19] (Fig. S2). MMP^3^C v2 exhibited superior discriminative performance to MMP^3^C v1 in pan-cancer datasets (Fig. S2A). Single-sample gene set enrichment analysis (ssGSEA) [20] further confirmed the presence of the Warburg effect across pan-cancer cohorts (Fig. S2B). Collectively, these findings indicate that the MMP^3^C v2 framework offers enhanced discriminative power in capturing RA-specific metabolic rewriting.

#### Application and discovery of RA-specific metabolic alterations

MMP^3^C v2 analysis revealed upregulation of glycolysis and the PPP in RA (Fig. 2c and d), consistent with enhanced biosynthetic and energetic demands in inflammatory conditions [21, 22]. The activation of PPP also showed a modest relative increase compared with glycolysis (Fig. S1B). In contrast, inositol phosphate metabolism was markedly attenuated, suggesting reduced signaling capacity through this metabolic axis (Fig. 2c and d). Subsequent SHAP contribution analysis revealed that HK3, PGK2 (key glycolytic enzymes), and ATP-related genes made major contributions to the observed metabolic shifts (Fig. 2d). Then, a meta-analysis across five case–control cohorts from the ComBat-integrated dataset, before ComBat batch correction, was conducted to identify reproducible PMP signatures associated with RA (Fig. 2e). This analysis revealed a set of significantly dysregulated pathway pairs, which were integrated into a global metabolic interaction network (Fig. 2f). Network topology analysis identified the top 10 hub metabolic pathways—including fatty acid degradation, folate biosynthesis, and nicotinate and nicotinamide metabolism—that exhibited strong connectivity and consistent alterations in RA [23–25], underscoring their important roles in RA pathogenesis.

### Evaluation of candidate PMP biomarkers for RA diagnosis and comparison with published reports

**Figure 3:**
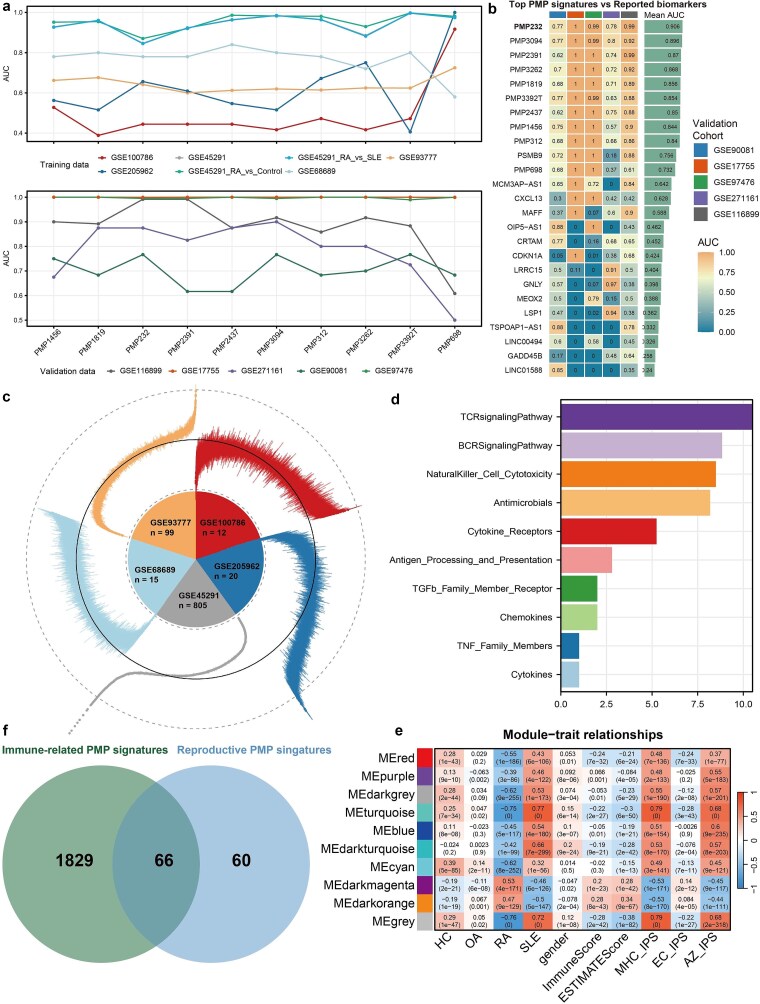
Benchmarking of single PMP biomarkers for RA against established biomarkers and identification of immune-related, reproducible PMP signatures for RA diagnosis. (a) Line plots displaying the AUC values of the top 10 PMP biomarkers across multiple original training datasets (batch effect retained) and independent validation datasets. (b) Heatmap illustrating the AUC values of the top 10 PMP signatures, compared with previously reported biomarkers, across five independent validation cohorts. (c) Pie chart illustrating the meta-analysis results derived from five original training datasets without batch-effect correction. (d) ImmuPagerank method identifying PMP patterns significantly associated with immune pathways. (e) WGCNA analysis revealing the association between metabolic plasticity modules and immune phenotypes computed by ESTIMATE and IPS algorithms. (f) Venn diagram showing the overlap between immune-related PMP signatures and cross-cohort meta-analysis-derived signatures.

Building upon our previously established computational framework [16, 26, 27] that revealed metabolic shift as a hallmark of disease, we next systematically evaluated the diagnostic potential of sample-level PMP interaction signatures in RA. First, we evaluated the discriminative ability of all 6960 PMP interaction signatures for distinguishing RA from healthy and disease controls (e.g. SLE and OA) using the ComBat-integrated dataset. Candidate signatures were then validated based on AUC values across validation datasets to identify robust biomarkers (Table S4). Remarkably, the top-performing PMP signatures demonstrated consistent discriminatory power across five research datasets from the ComBat-integrated dataset (batch effects were deliberately retained; Fig. 3a), as well as five independent validation cohorts (Fig. 3b).

Furthermore, comparative analyses between these top 10 PMP signatures and previously published messenger RNA (mRNA) and long non-coding RNA (lncRNA) biomarkers (e.g. CXCL13, PSMB9, and LINC00494; Table S5) revealed superior predictive performance of the PMP-based signatures in stratifying RA versus non-RA patients across five validation datasets (Fig. 3b). Notably, one PMP signature (PMP232; PPP–Nicotinate and nicotinamide metabolism) achieved outstanding mean AUC values of 0.935 across five independent validation datasets (Fig. 3b). These results demonstrate that PMP-based biomarkers achieve high diagnostic accuracy across heterogeneous cohorts and outperform previously reported mRNA/lncRNA biomarkers.

### Integrative identification of immune-related metabolic plasticity features for the RA diagnostic model

To improve RA diagnostic accuracy and generalizability, we integrated multiple PMP signatures and tested 110 machine learning model combinations to construct a robust diagnostic model. Initially, we used our meta-analysis-based feature selection pipeline to obtain reproducible PMP signatures across multiple cohorts and technological platforms. We performed differential PMP analysis (RA versus non-RA) across five research datasets from the ComBat-integrated cohort before batch correction (Fig. 3c). Using the “limma” R package [25] (adjusted for age and sex), we identified differential PMP signatures and subsequently integrated the results (adjusted *P* value <.05, I2 < 50; Table S6) through meta-analysis via the “metafor” R package [26].

After evaluating the predicting performance of individual PMP features, we specifically focused on immune-related PMP features, rather than all metabolic features, because RA is increasingly recognized as a prototypical immunometabolic disease in which metabolic rewiring directly influences immune-cell activation, cytokine production, and persistent synovial inflammation [28–30]. Accordingly, we identified immune-metabolic features based on the ImmuPagerank algorithm adapted from the ImmLnc framework [31] (Supplementary Methods and Fig. 3d) and weighted gene co-expression network analysis (WGCNA; Fig. 3e). We employed the ImmuPagerank method to identify immune-metabolic plasticity features implicated in RA, suggesting coordinated remodeling of amino acid utilization and lipid metabolic homeostasis during immune activation (Fig. 3d and Table S7). Immune-related traits were quantified using the Estimation of STromal and Immune cells in MAlignant TIssue using Expression data (ESTIMATE) and Immunophenoscore (IPS) algorithms and integrated into the WGCNA analysis. Subsequently, PMP modules showing significant correlations (|Pearson r| > 0.5, *P* < .05) with RA disease status, ImmuneScore, MHC_IPS, or AZ_IPS were retained (Fig. 3e and Table S8). The trait-associated modules identified by WGCNA, together with immune-related PMP features identified by ImmuPagerank, were carried forward for downstream analyses. Ultimately, 126 cross-platform reproducible PMP features (selected by meta-analysis) were combined with the 1895 immune-related signatures to train and evaluate 110 machine learning pipelines (Fig. 3f).

### The development and validation of an RA diagnostic model based on immune-related metabolic plasticity signatures

**Figure 4:**
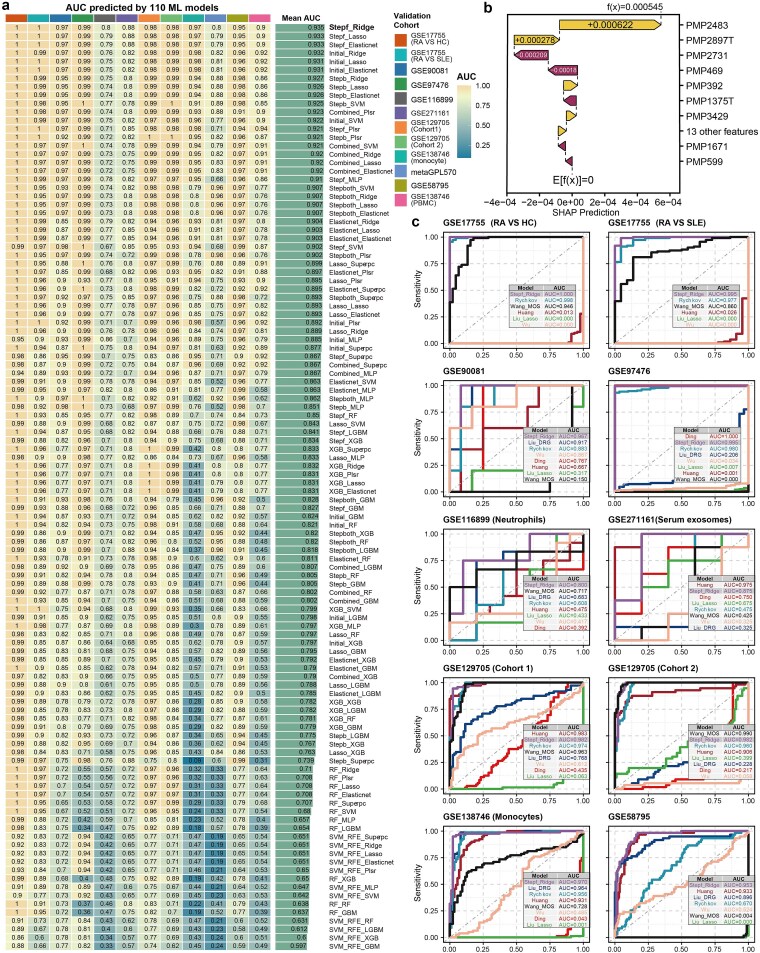
Establishment and validation of an RA diagnostic model based on 110 machine learning model combinations. (a) AUC values predicted by all 110 models across 12 independent validation cohorts. (b) SHAP analysis revealing the importance of PMP signatures used in the optimal machine learning model. (c) ROC curves comparing StepfRidge with seven previously published models on the validation datasets.

A total of 66 overlapped features identified in previous analyses were subjected to a rigorous filtering pipeline based on their association with RA outcomes, assessed using Pearson correlation coefficients and AUC values, yielding 44 retained features (AUC > 0.6 and |r| > 0.5; Table S9). To identify the most robust diagnostic architecture, we systematically explored the integration of 12 machine learning and deep learning algorithms, resulting in 110 distinct hybrid model combinations based on Python packages (scikit-learn [32] and Optuna [33]). Specifically, stepwise regression, Least Absolute Shrinkage and Selection Operator (LASSO), ridge regression, elastic net, Random Forest (RF), eXtreme Gradient Boosting (XGBoost), and Support Vector Machine-Recursive Feature Elimination (SVM-RFE) were used to further filter important features in the ComBat-integrated dataset. These selected features were then fed into diverse machine learning and deep learning classifiers. By pairing the feature selection methods with the classification algorithms, we constructed 110 distinct hybrid model pipelines. The optimal hyperparameters for each predicting pipeline were determined by employing five-fold cross-validation, and the corresponding final models were fitted to the training dataset using these settings (Figs 4a and 1d; details in Supplementary Methods) [34, 35]. Among these, the integrated stepwise forward–ridge regression (StepfRidge) model with 22 selected features yielded a mean AUC of 0.936 across 12 independent validation datasets spanning microarray and next-generation sequencing (NGS) platforms (Fig. 4a and b). The 12 validation datasets comprised 6 case–control cohorts and 6 RA-only cohorts, the latter of which were supplemented with healthy control data sourced from matching platforms and processed using an identical pipeline to avoid batch effect (Table S3). To prevent directionality bias in AUC calculations, we aligned predicted probabilities with RA labels consistently across all training and validation datasets.

**Figure 5:**
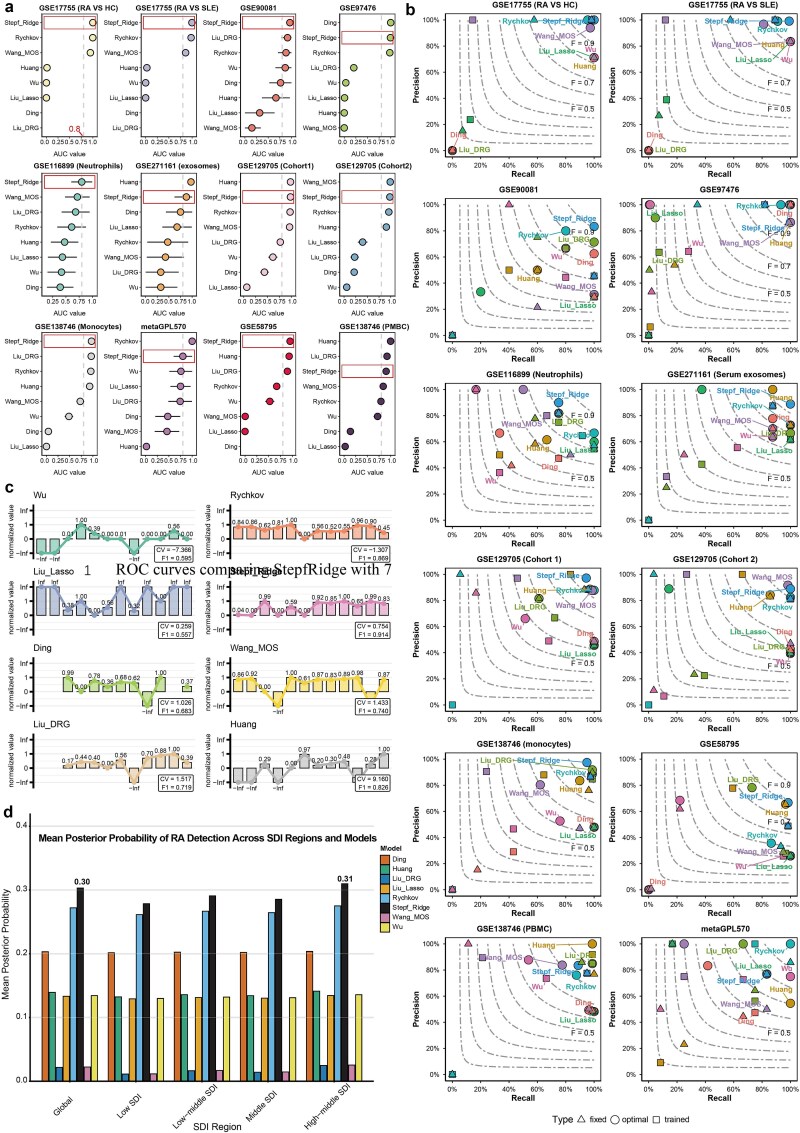
Comparison of the StepfRidge model with previously published RA diagnostic models. (a) AUC values predicted by the StepfRidge model and seven published diagnostic models across various independent validation cohorts. (b) The distribution of F1 scores for each model across independent validation cohorts under three predefined decision thresholds. (c) Comparison of optimal cutoff values across various diagnostic models for diverse independent validation cohorts. (d) Clinical screening simulation of diagnostic models based on the BPPI across different RA prevalence levels derived from the GBD database.

We comprehensively compared the StepfRidge model with seven previously published RA diagnostic models (Table S5). Our model achieved a superior AUC of 0.936, exceeding that of the second-best model (Rychkov’s model [36]; mean AUC = 0.856; Figs 4c and 5a). Notably, it also distinguished RA from SLE with perfect accuracy (AUC = 0.995 in the GSE17755 cohort, *n* = 133). The model performed well across diverse biological contexts, including neutrophils (AUC = 0.80 in GSE116899, *n* = 22) and extracellular vesicles (AUC = 0.875 in GSE271161, *n* = 14), and maintained strong efficacy in pregnant (GSE235508) and medication-treated (GSE15573 and GSE229449) cohorts (Figs 5a and S3). Then, we compared F1 scores across predefined decision thresholds [the optimal (maximizing F1), the training-derived, and a fixed threshold of 0.5] with those of published RA diagnostic models. Figure 5b shows the distribution of F1 scores under these threshold settings across independent validation datasets. The StepfRidge model maintained consistently higher F1 scores and showed remarkable stability (mean optimal F1 score = 0.914) with the smallest coefficient of variation (Fig. 5b and c). To further evaluate clinical utility, we calculated the Bayesian posterior probability index (BPPI) [37, 38] for various models across different assumed disease prevalence levels, derived from the 2023 Global Burden of Disease (GBD) database (Supplementary Methods). Figure 5d presents the BPPI values across plausible RA prevalence scenarios, reflecting the prevalence-adjusted positive predictive value of each model, indicating that StepfRidge consistently achieved higher posterior probabilities across all prevalence levels. Collectively, these results highlight that the StepfRidge model delivers both high discriminative power (AUC) and robust decision performance (F1 score), underscoring its reliability and potential utility in diverse clinical scenarios.

### Cross-tissue scRNA-Seq analysis of metabolic reprogramming in the RA microenvironment

**Figure 6:**
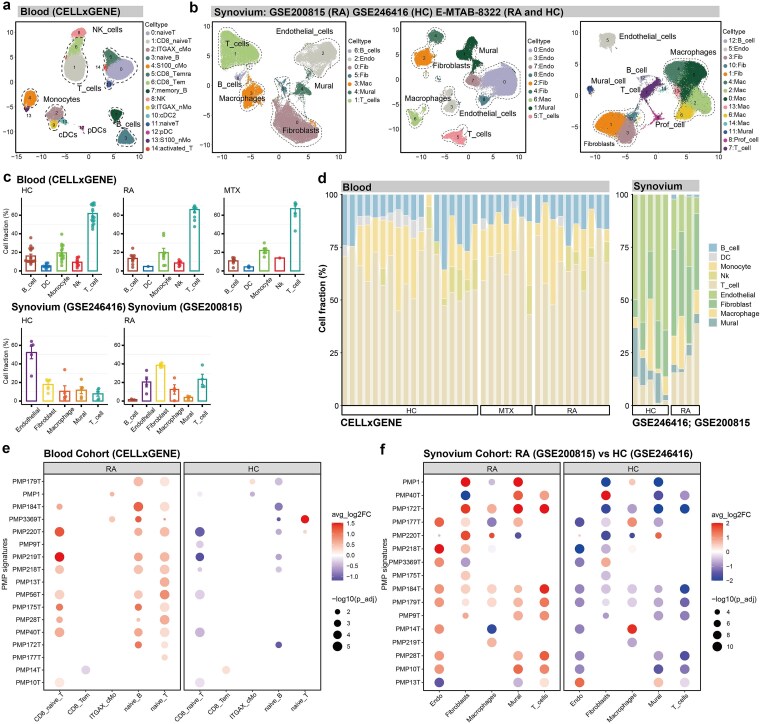
Identification of PMP interaction patterns at the single-cell resolution. (a) UMAP plot of 74 779 cells sampled from the peripheral blood of RA patients and healthy controls, showing 6 major cell types. (b) UMAP plot of the synovium single-cell RNA-seq data for three cohorts: GSE246416 (RA patients), GSE200315 (healthy controls), and E-MTAB-8322 (includes multiple disease states). (c) Bar plots showing the distribution of cell types in the blood and synovial microenvironments. (d) Stacked bar plots illustrate the proportions of these cell types across various disease states. Heatmaps showing differential PMP interaction patterns in the immune microenvironment between untreated RA patients and healthy controls in peripheral blood (e) and synovial (f) tissues.

To resolve cell-type-specific PMP interaction patterns underlying RA, our framework MMP^3^C v2 was used to analyze single-cell RNA-seq datasets from peripheral blood and synovial tissues. To reduce single-cell dropout, missing metabolic gene expression values were imputed using DeepImpute [37]. In peripheral blood (sourced from the CELLxGENE database), clustering analysis of 74 779 cells from RA patients and healthy controls revealed immune lineages including T cells [naive, activated, CD8+ naive, CD8+ effector memory (Tem), and CD8+ terminally differentiated effector memory (Temra) cells]; B cells (naive and memory); natural killer (NK) cells; dendritic cells [plasmacytoid (pDCs) and classical (cDCs)]; and monocytes [classical (cMo) and non-classical (nMo), alongside their respective S100A8+ and ITGAX+ subsets; Figs 6a and S4]. In the synovium, UMAP embeddings revealed cell populations including T cells, B cells, macrophages, fibroblasts, endothelial cells, and mural cells from three independent cohorts (GEO: GSE246416, GSE200815, and ArrayExpress: E-MTAB-8322; Figs 6b and S4–S5). Notably, we observed significant B-cell infiltration in the synovial tissue of RA patients (Fig. 6c and d).

Differential PMP analysis revealed RA-specific metabolic alterations (Fig. 6e and f). In peripheral blood, Tem exhibited a pronounced glycolytic shift, with elevated glycolysis and attenuated OXPHOS (Fig. 6e). Concurrently, naïve B cells from RA patients exhibited reduced vitamin B6 metabolism, suggesting dysregulation of biosynthetic and cofactor pathways (Fig. 6e). In the RA synovium, macrophages displayed a Warburg-like phenotype, characterized by enhanced glycolysis and PPP activity, with reduced OXPHOS activity (Fig. 6f). Together, these findings demonstrate that RA-associated metabolic rewiring is cell-type- and tissue-specific.

### Trajectory analysis of infiltrating T cells reveals fate-linked metabolic reprogramming in RA

**Figure 7:**
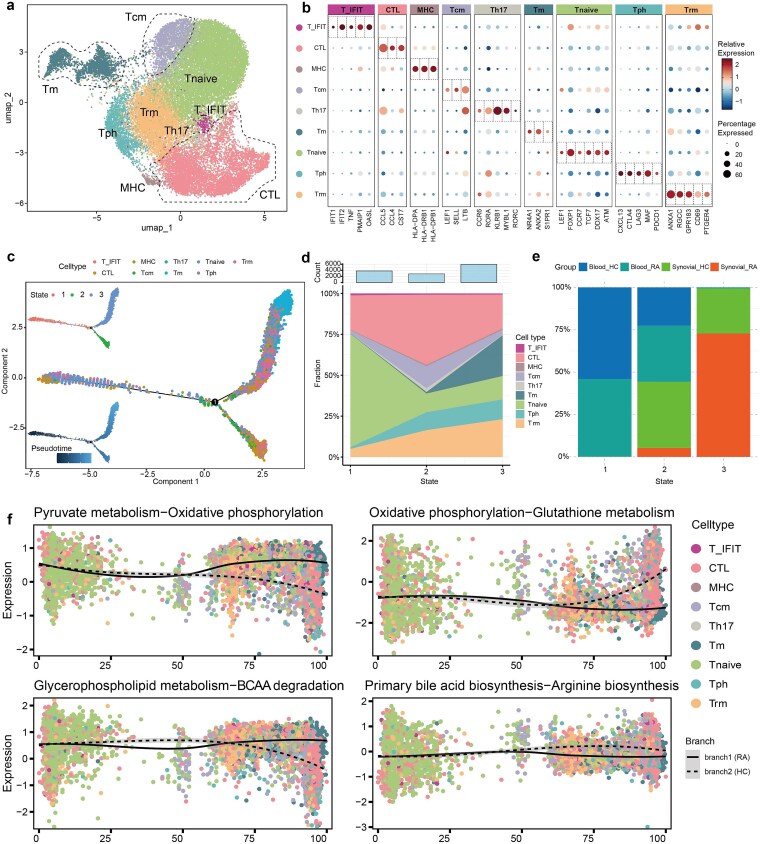
Identification of fate-associated PMP interaction patterns by cross-tissue T cells analysis and trajectory analysis. (a) UMAP plot of 12 444 T cells in RA patients and healthy controls from peripheral blood and synovium tissues displaying 10 T cell subtypes. (b) Dot plot of significant markers for each T cell subtype. (c) Cross-tissue trajectory analysis of all T cells identifying two distinct cell fates. (d) Stacked percentage chart revealing the distribution of T cell subtypes in various trajectory states. (e) Percent bar plot revealing the distribution of T cells from different disease states and tissue types in various trajectory states. (f) Dot plots of dynamic activity difference of PMP interaction patterns along two cell fates (RA versus HC).

T cell activation is intricately linked to metabolic reprogramming, which is essential for supporting their effector functions and differentiation [38]. To explore how PMP interaction patterns affect T cells’ function, T cells were extracted from individual datasets (CELLxGENE, GSE246416, GSE200815, and E-MTAB-8322), merged, and subsequently subjected to batch effect correction. All 12 444 T cells were initially downsampled using Seurat’s sketching method [39] to account for unequal cell distributions across tissues and disease states. This step was repeated to assess robustness (Fig. S6). The T cells were categorized into diverse subpopulations using UMAP, including naïve T cells, central memory T cells (Tcm), tissue-resident memory T cells (Trm), memory T cells (Tm), T helper 17 (Th17) cells, T peripheral helper (Tph) cells, cytotoxic T lymphocytes (CTLs), interferon-responsive (IFIT+) T cells, and MHC-expressing T cells (Fig. 7a and b). Then, we performed trajectory analysis, which segregated those T cells into two major fates, distinguishing RA from healthy controls (Fig. 7c–e). Pseudotemporal analysis further revealed the differential representation of naïve, effector, and regulatory-like states along these branches (Fig. 7d).

We employed MMP^3^C v2 to quantify PMP dynamics along the T-cell trajectories, revealing fate-specific metabolic plasticity. Cells on the RA-associated fate showed increased PMP activity along pyruvate metabolism → OXPHOS axis (Fig. 7f), consistent with tighter coupling between central carbon metabolism and mitochondrial respiration in RA-linked T cells [39, 40]. Similar shifts emerged across glucose metabolism (glycolysis, TCA cycle, and glutathione metabolism; Fig. S7). In contrast, glycerophospholipid metabolism → branched-chain amino acid (valine, leucine, isoleucine) degradation axis showed the opposite trend (Fig. 7f). RA-fated cells exhibited a preference for increased activity in arginine biosynthesis over primary bile acid synthesis (Fig. 7f). These PMP alterations varied with RA disease state, suggesting microenvironmental modulation of metabolic interaction patterns in RA.

### Metabolic plasticity driven by GWAS-disk genes remodels the RA immune microenvironment

**Figure 8:**
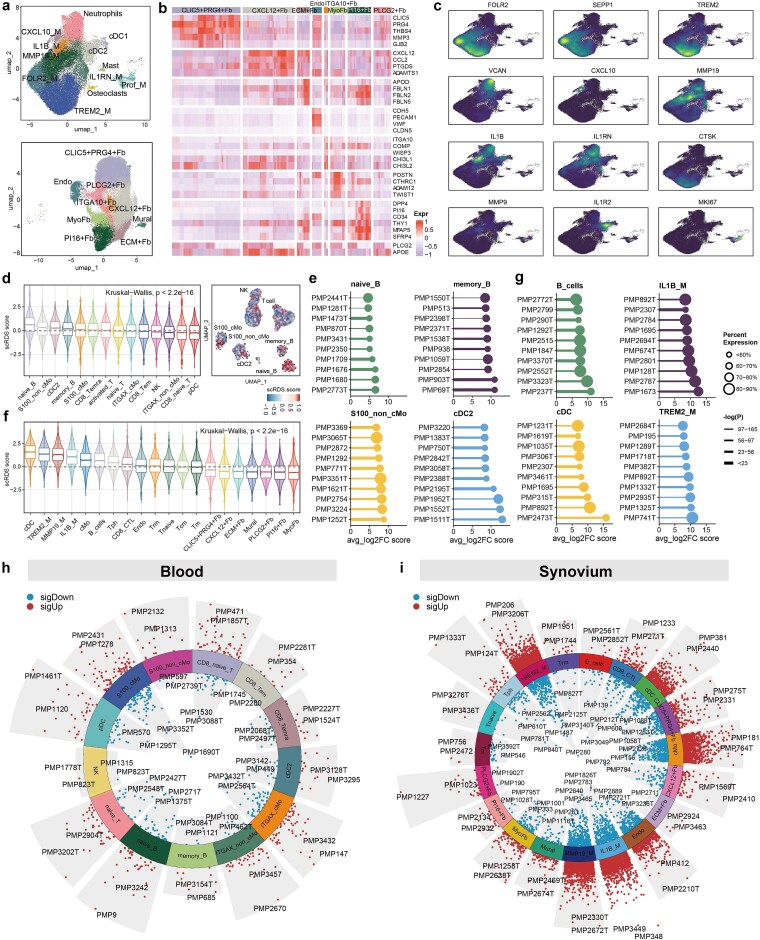
Identification of GWAS-risk associated with PMP interaction patterns by integrating scDRS and MMP^3^C analysis. (a) UMAP plot of RA patients and healthy controls from peripheral blood and synovium tissues displaying fibroblast and macrophage compartments. (b) Heatmap of significant markers of each fibroblast cell subtype. (c) UMAP plots displaying various cell subtypes of myeloid cells. (d) Violin plots display the association of RA with various cell types in the blood microenvironment. (e) Violin plots display the association of RA in various cell types in synovial microenvironments. (f) Lollipop plots display the significance of PMP signatures in specific cell types in the blood microenvironment. (g) Lollipop plots display the significance of PMP signatures in specific cell types in the synovial microenvironment. Volcano plot shows differential PMP interaction patterns analysis stratified by cell subtype in (h) peripheral blood and (i) synovium.

Building upon the established paradigm that somatic and germline genetic alterations can act as fundamental drivers of metabolic reprogramming [41]. We examined cell-type-specific PMP interaction patterns linked to genetic risk loci across multiple cohorts, including peripheral blood (from the CELLxGENE database) and synovial tissues (GEO: GSE246416 and GSE200815; ArrayExpress: E-MTAB-8322). Initially, UMAP embeddings resolved discrete fibroblast populations [e.g. CXCL12^+^, PLCG2^+^, ITGA10^+^, PI16^+^, and CLIC5^+^PRG4^+^ fibroblasts; myofibroblasts (MyoFb); ECM-producing fibroblasts (ECM-Fb); endothelial-associated fibroblasts (Endo-Fb); and mural cells] and macrophage/myeloid states [e.g. FOLR2^+^, IL1B^+^, MMP19^+^, TREM2^+^, IL1RN^+^, and CXCL10^+^ macrophages; proliferating macrophages; classical dendritic cells (cDC1, cDC2); mast cells; osteoclasts; and neutrophils; Fig. 8a–c]. We quantified the cell-level disease relevance using the single-cell disease relevance scoring (scDRS) algorithm [42], based on RA genome-wide association study (GWAS) summary statistics from the RABC database [43]. Notably, B cells and myeloid cells exhibited strong correlations with RA relevance scores both in blood and synovium (Fig. 8d–g). These findings were further validated based on an independent GWAS dataset (GCST90044540) from the RABC database (Fig. S8).

We next performed cell-type–stratified differential analyses using per-cell PMP interaction scores (see Supplementary Methods for details), revealing enriched pathway pairs, such as those involved in glucose metabolism and nicotinate/nicotinamide metabolism in peripheral blood memory B and nMo populations, and Pentose phosphate pathway-One carbon pool by folate in synovial B cells (Fig. 8f and g). To investigate PMP signatures linked to high genetic risk, we stratified cells into high- and low-risk groups based on scDRS score quartiles. In peripheral blood, most cell types exhibited modest PMP shifts with few significant pathway pairs (Fig. 8h), whereas the synovium showed pronounced changes concentrated in fibroblasts and macrophages (Fig. 8i). Notably, several top-ranked PMP pairs in synovial fibroblasts and macrophages involved glucose metabolism (e.g. TCA → OXPHOS) and lipid/branched-chain amino-acid axes, implying enhanced metabolic crosstalk in the RA joint (Fig. 8i).

### Integrated cell–cell communication and MMP^3^C analyses uncover ligand- and receptor-associated metabolic plasticity patterns

**Figure 9:**
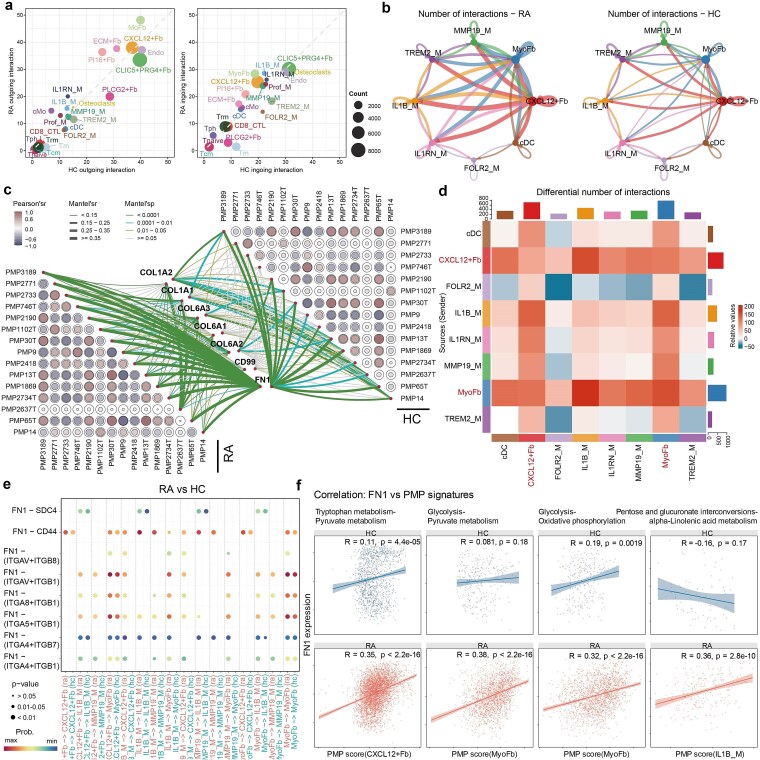
Integrating analysis of cell–cell communication and MMP^3^C v2 reveals ligand- and receptor-associated PMP interaction patterns. (a) Dot plot showing significantly altered cell subtypes involved in intercellular communication between RA patients and healthy controls. (b) Inferred cell–cell signaling networks in RA patients and healthy controls. (c) Correlation analysis revealing the PMP interaction patterns associated with the FN1 ligand in RA patients and healthy controls. (d) The heatmap displaying FN1-related signaling among each cell type in RA compared with healthy controls. (e) Dot plot showing differentially expressed ligand–receptor pairs between RA patients and healthy controls across various cell subtypes. (f) Correlation analysis illustrating FN1-associated PMP interaction patterns across multiple cell types in the RA immune microenvironment.

Beyond cell-intrinsic functions, metabolic reprogramming acts as a critical driver that actively remodels the intercellular communication [44]. We next examined which metabolic reprogramming might mediate alterations of cell–cell communications in RA. At first, a global cell–cell signaling network was established using CellChat [45] based on the synovial cohorts (GSE246416, comprising five healthy controls and GSE200815, comprising four RA patients; Fig. 9a and b). Comparative analysis between RA and healthy synovium revealed several cell populations (e.g. MyoFb, CXCL12^+^ Fb, and IL1B^+^ M) exhibiting markedly altered incoming/outgoing signaling patterns (Fig. 9b). Among the top 200 dysregulated ligands, an FN1-centered signaling module emerged as a dominant hub, with COL1A2, COL1A1, and CD99 playing key roles in enhancing RA communication (Fig. 9c and d and Table S10). FN1-centered signaling was predominantly contributed by MyoFb and CXCL12^+^ fibroblasts, with the most intense communication occurring between MyoFb and IL1B^+^ macrophages via FN1–integrin (ITGAV, ITGB1, ITGB8) interactions (Figs 9d, e, and S9A).

Pearson correlation analysis demonstrated that the strength of FN1-mediated communication was significantly correlated with PMP interaction patterns involving glycolysis–pyruvate and glycolysis–OXPHOS (Fig. 9c and f). Notably, these correlations were cell-type specific: inflammatory MyoFb and CXCL12^+^ fibroblasts, and IL1B+ M cells exhibited positive correlations between FN1 expression and glucose- and energy-related PMP pairs, whereas such associations were absent in healthy controls (Fig. 9f). In contrast, FOLR2^+^ M2-like macrophages in RA synovium exhibited loss of this correlation (Fig. S9B). Furthermore, we performed virtual knockout of FN1 in MypFb cells from RA using the “scTenifoldKnk” Python package [46]. GSEA exhibited that virtual knockout perturbed genes enriched in the OXPHOS (adjusted *P* value = .005) and glutathione metabolism (*P* value = .02) pathways (Fig. S10 and Table  S11). Together, these findings highlighted FN1-centered cell–cell communication as a pivotal signaling axis associated with metabolic reprogramming in the RA synovium, which strongly correlates with alterations in glucose metabolism and cellular energy pathways.

## Discussion

We present MMP^3^C v2, a network-aware framework for quantifying directed PMP interaction patterns in RA. Applied to bulk and single-cell cohorts, MMP^3^C v2 revealed consistent RA-specific metabolic rewiring. These observations motivated us to evaluate whether PMP patterns could serve as reliable diagnostic features, addressing the challenges of limited sensitivity, specificity, and cross-cohort reproducibility in existing RA biomarkers. By integrating per-sample PMP scoring, network prioritization (via WGCNA and ImmuPagerank), and rigorous feature selection via meta-analysis across five research cohorts, we identified a compact and cross-platform set of immune-metabolic features. These features underpinned an ensemble StepfRidge diagnostic model, which achieved robust performance (mean AUC = 0.936 and mean F1 score = 0.915; Fig. 5a–c) across 12 independent validation cohorts.

A key methodological innovation lies in our feature selection strategy: metabolic signatures were identified through meta-analysis across five training cohorts before batch correction. This approach prioritizes signals robust to technical variation, rather than relying solely on *post-hoc* harmonization with the ComBat algorithm, which likely underlies the model’s exceptional cross-platform stability. The distinguishing ability of our model between RA and SLE indicates that the identified immune-metabolic signatures capture disease-specific processes rather than generic inflammation. This specificity may prove valuable for early diagnosis in seronegative or overlap syndrome cases. Notably, the model also performed well across heterogeneous sample types, including neutrophils and extracellular vesicles, indicating that RA-associated metabolic dysregulation is systemically imprinted and detectable beyond conventional PBMC assays. Our model demonstrates the feasibility of less invasive liquid biopsy approaches for RA diagnosis.

Additionally, the strong signal in EVs suggests they carry disease-relevant metabolic information, supporting their emerging role as intercellular metabolic messengers in autoimmunity [47, 48]. WGCNA analysis revealed immune-metabolic signatures enriched in amino acid, sterol, and glycerophospholipid metabolism that strongly correlated with MHC-related immune scores. This association is mechanistically coherent: MHC antigen presentation requires amino acids for peptide loading and membrane lipids for vesicular trafficking and immunological synapse formation [49, 50]. The coordinated upregulation of these metabolic pathways may represent a metabolic “preparedness state” that supports heightened immune surveillance in RA [51, 52].

At single-cell resolution, distinct metabolic phenotypes were observed across cell types and tissue compartments. Synovial macrophages displayed a Warburg-like metabolic signature (elevated glycolysis and PPP, suppressed OXPHOS), consistent with the rapid ATP generation and biosynthetic precursor supply required for sustained cytokine production and phagocytic activity [29, 53]. This metabolic shift is a hallmark of inflammatory macrophages across various disease contexts and represents a potentially druggable vulnerability [54]. The glycolytic shift in peripheral effector memory T cells extends this paradigm beyond the synovium, suggesting that metabolic reprogramming in RA is systemic, not just localized. Integration analysis of single-cell transcriptomics with GWAS data identified blood B cells as a genetically associated cell type in RA, exhibiting metabolic plasticity in glucose and nicotinamide metabolism (Fig. 8d–f). The nicotinamide pathway is crucial for NAD+ biosynthesis, a critical cofactor for sirtuin-mediated epigenetic regulation and redox homeostasis [55]. An association between dysregulated NAD+ metabolism and metabolic stress frequently accompanies aberrant B cell activation and autoantibody production in RA [56]. Our cell–cell communication analysis uncovered FN1-centered signaling modules whose activity correlated with specific metabolic programs (elevated glycolysis coupled with suppressed pyruvate metabolism and OXPHOS) within pathogenic cell subpopulations (Fig. 9c and f). These findings suggest that FN1-mediated ECM remodeling may contribute to a potential feedforward coupling with metabolic reprogramming in RA synovium. Previous studies suggest that Fibronectin binding to integrins can activate PI3K/AKT/mTOR signaling, a key regulator of aerobic glycolysis [57, 58]. Importantly, the virtual knockout of FN1 in MyoFb cells was associated with significant perturbation of genes enriched in OXPHOS (adjusted *P* value = .005) and glutathione metabolism (*P* value = .02) pathways, supporting a role for FN1 as a crucial regulator linking ECM remodeling to metabolic adaptation. The concurrent glycolytic rewiring and FN1-mediated matrix remodeling identified in our study likely represent a coordinated pathogenic adaptation to severe environmental stresses within the RA synovium, such as hypoxia and oxidative stress [29, 59, 60]. This reprogrammed metabolism likely provides essential biosynthetic substrates for further FN1 synthesis; for instance, enhanced glycolytic flux feeds the hexosamine biosynthetic pathway to supply UDP-GlcNAc for FN1 glycosylation [61, 62], thereby creating a robust feedforward loop. Ultimately, this dynamic interplay between environmental pressure, metabolic plasticity, and extracellular matrix signaling may perpetuate chronic synovial inflammation [63]. Disrupting this loop through targeting ECM-integrin interactions or downstream metabolic effectors may offer a novel therapeutic avenue [64].

Key strengths of our study include: (i) a topology-aware metric that leverages PPI shortest-path distances to weight expression differences and thus prioritize biologically plausible cross-pathway links; (ii) a dual-validation strategy employing batch-corrected data for model development alongside cohort-level meta-analysis of batch-effect–retained data for feature selection, ensuring the identification of platform-robust signals; and (iii) validation at single-cell resolution that localizes PMP changes to specific cell types and trajectories.

The limitations of this study include the following. First, transcriptomic divergence and network proximity serve as proxies for metabolic activity and do not directly measure enzymatic activity, metabolite concentrations, or true metabolic flux; hence, our expression-based PMP scores should be interpreted as hypothesis-generating rather than as conclusive evidence of altered metabolic flux. Second, the PPI network is incomplete and biased toward well-studied proteins; choices in network topology can influence PMP estimates. Third, although we employed rigorous meta-analysis and cross-cohort validation, residual confounders (medication, disease duration, comorbidities) may contribute to observed differences. However, we adjusted for age and sex; unmeasured clinical variables could still be a potential source of heterogeneity. Finally, regarding clinical implications, while our StepfRidge diagnostic model achieved high robustness across 12 independent cohorts, translating a multi-gene transcriptomic signature into a routine clinical assay presents practical challenges, such as the cost of RNA sequencing and the need for standardized targeted panels. Future prospective clinical trials in real-world clinical settings are required to fully evaluate the cost-effectiveness and clinical utility of the MMP^3^C v2 framework for early RA diagnosis and patient stratification.

In summary, MMP^3^C v2 provides a scalable, network-aware approach for mapping pairwise metabolic plasticity. Applied to RA, it reveals a consistent map of immune-linked metabolic rewiring across resolutions and delivers a diagnostic model that is robust across cohorts and platforms, addressing a central unmet need in RA diagnosis. The identified PMP signatures combine biological interpretability with cross-cohort diagnostic robustness, offering a translationally relevant resource for mechanistic studies and potential biomarker development in RA and related immune-metabolic disorders.

## Materials and Methods

### Data collection

Raw transcriptomic data and preprocessed data were obtained from the Gene Expression Omnibus (GEO) database [65] and Rheumatoid Arthritis Bioinformatics Center (RABC; http://www.onethird-lab.com/RABC/) [43], encompassing mRNA microarray datasets and RNA sequencing (RNA-seq) datasets (Table S3). The scRNA-seq datasets were retrieved from public repositories. The CELLxGENE dataset includes 18 RA and 18 healthy control peripheral blood samples, and 2 synovial tissue scRNA-seq datasets (GSE246416 and GSE200815) were downloaded from the GEO. Additionally, the ArrayExpress dataset E-MTAB-8322 was downloaded from the ArrayExpress database. Sample accession numbers and detailed sample metadata are listed in Table S12. The full data processing and analysis workflow is detailed in the Supplementary Methods.

### Establishment of the directed PMP interaction scores

Based on our previous work [16, 26], we developed MMP^3^C v2 to quantify directed PMP interaction scores by integrating gene expression with PPI network topology [17]. Using 84 KEGG metabolic pathways, we computed directed PMP interaction scores for all ordered pathway pairs, yielding 6960 directed comparisons. For each ordered pathway pair MP_a_ ➔ MP_b_, we computed a per-sample directional score S(a➔b) that aggregates gene-level expression differences between genes in MP_a_ and their nearest neighbors h in MP_b_ on the PPI graph, weighing each contribution by the reciprocal of the shortest distance between gene and network.

#### Directional PMP score—formal definition

For each gene g ∈ MP_a_, we define the network distance between g and MP_b_ on the PPI network as follows:


$${h}_g=\arg\ \underset{h\in{MP}_b\ }{\min }d\left(g,h\right)$$

Where d(u,v) is the shortest-path distance between node u and v within the MP_b_ subnetwork of the PPI graph. Gene expression values were transformed using log2(TPM + 1) to mitigate biases arising from gene length and sequencing depth. For each sample (or cell), the resulting normalized expression for gene g is denoted as Eg(.). The per-sample directed PMP score from MP_a_ to MP_b_ was computed by, for each gene in MP_a_, taking the expression difference between the gene and its nearest neighbor in MP_b_ along the PPI graph, weighed by the corresponding shortest-path length; these weighed differences were then summed to obtain the final interaction score.


$$P\mathrm{MP}\ {\mathrm{score}}_{a\to b}^s=\sum_{s\in{MP}_a}\frac{E_s(source)-{E}_s(target)}{d\left( source,\kern0.5em b\right)}$$

To avoid zero-distance artifacts, genes that belong to both MP_a_ and MP_b_ are, for the directed calculation MP_a_➔MP_b_, treated as members of the source MP_a_ and excluded from the target set MP_b_. This assignment is applied directionally (the reverse direction MP_b_➔MP_a_ treats shared genes as members of MP_b_). Pathway pairs in which one pathway is entirely contained within the other were excluded from analysis (see Table S2).

#### Per-sample normalization for cross-sample comparisons

After computing the directed score ${S}_{a\to b}^s$ for every ordered pathway pair and sample, we assembled each sample’s PMP interaction score vector:


$${v}_s=\left({S}_{1\to 2}^{(s)},{S}_{1\to 3}^{(s)},\dots, {S}_{83\to 84}^{(s)}\right)$$

To make PMP profiles comparable across samples while preserving relative pairwise patterns, we applied cosine (L2) normalization to each sample vector:


$${v}_s^{`}=\frac{v_s}{\sqrt{\sum_{a\ne b}{\left({S}_{a\to b}^{(s)}\right)}^2}}$$

**Key Points**

- We analyzed bulk transcriptomes from ~3400 individuals across multiple cohorts (RA, healthy controls, and disease controls, including osteoarthritis and systemic lupus erythematosus) and interrogated ~228 000 single cells to identify metabolic-plasticity signatures and cell-type-specific metabolic rewiring.
- Our metabolic-plasticity signatures outperform 23 reported mRNA/lncRNA biomarkers in multiple independent validation datasets. Using an integrated discovery cohort (*n* = 2350), we trained 110 machine learning models and validated them across 12 independent cohorts (*n* = 1113), achieving robust generalizability (mean AUC >0.93, mean F1 score >0.91) and surpassing seven published diagnostic models.
- At single-cell resolution, MMP^3^C v2 unveils key metabolic alterations associated with RA pathogenesis, e.g. glucose, fatty acid, glutathione, and folate metabolism, and incorporates a regression module to nominate candidate driver genes for experimental validation.

## Supplementary Material

Supplementary_material_bbag307

## Data Availability

All real datasets used within this study are retrievable from public databases with details provided within the Supplementary Information. All the source code for reproducing the examples presented in this paper and the “mmp3c” R package can be consulted in the following GitHub repository: https://github.com/ChenHuangMUST/mmp3c.git.
