# Supplementary material for: MMP3C v2: a network-based framework decoding metabolic plasticity in rheumatoid arthritis, enabling accurate diagnosis and uncovering cell-type-specific metabolic rewiring
Source: Brief Bioinform. 2026 Jun 14;27(3):bbag307. doi: 10.1093/bib/bbag307 (PMC13264964; doi:10.1093/bib/bbag307)
Supplement: Supplementary_material_bbag307 [file supplementary_material_bbag307.zip › SF_revised_bbag307.pdf]

MMP^{3}C v2: a network-based framework decoding metabolic plasticity in rheumatoid arthritis, enabling accurate diagnosis and uncovering cell-type-specific metabolic rewiring

Xingyu Chen[1#], Zihan Wang[1#], Min Deng[2#], Jianxiang Huang[1], Naishu Zhang[1], Zheng Wu[1], Zelin Yi[1], Sangyu Li[1], Jiayue Qiu[1], Kit-Leong Cheong[3], Xin Chen[4,*], Chen Huang[1,*]

[1]Dr. Neher's Biophysics Laboratory for Innovative Drug Discovery, State Key Laboratory of Mechanism and Quality of Chinese Medicine & Faculty of Chinese Medicine, Macau University of Science and Technology, Taipa, Macao SAR 999078, China
[2]CRDA, Faculty of Health Sciences, University of Macau, Taipa, Macau SAR 999078, China
[3]College of Food Science and Technology, Guangdong Ocean University, Guangdong Provincial Key Laboratory of Aquatic Product Processing and Safety, Guangdong Province Engineering Laboratory for Marine Biological Products, Guangdong Provincial Engineering Technology Research Center of Seafood, Guangdong Provincial Engineering Technology Research Center of Prefabricated Seafood Processing and Quality Control, Zhanjiang City 524088, China
[4] School of Automation, Guangdong University of Technology, Guangzhou, 510006, China

*Corresponding authors.
E-mail address: chuang@must.edu.mo (C. Huang)
Telephone number: +853 6806 9981
Fax number: +853 2888 0022

E-mail address: xinchen@gdut.edu.cn (X. Chen)
Telephone number: +86 020 39322946
Fax number: +86 020 39322946
[#]These authors contributed equally to this work

Supplementary Methods

1. Cross platform gene expression profiles for RA, SLE, OA, and matched healthy controls

Raw and processed transcriptomic data were obtained from the Gene Expression Omnibus (GEO) database [1] and Rheumatoid Arthritis Bioinformatics Center (RABC; http://www.onethird-lab.com/RABC/) [2], encompassing mRNA microarray and RNA sequencing (RNA-seq) datasets (Table S3). For microarray datasets available in RABC, probe-level expression profiles were downloaded and mapped to official Entrez gene ID using platform-specific annotation files from GEO. Preprocessing of raw bulk microarray transcriptomic datasets followed the workflow described in the RABC database. Raw Affymetrix data were preprocessed using the Robust Multi-array Average (RMA) normalization method implemented in the ‘affy’ R package [3], whereas Agilent and Illumina data were processed with the neqc() function with default parameters in the ‘limma’ V3.64.3 R package [4]. Then, probes were systematically re-annotated to official Entrez gene ID based on platform-specific annotation files. For RNA-seq datasets obtained from GEO and RABC, raw count RNA-seq datasets were normalized to log2(transcripts per million [TPM] + 1) values. RNA-seq datasets normalized using other methods were converted to TPM when necessary. In addition, gene alias conflicts were resolved using the ‘mygene’ V1.44.0 R package [5] and GeneCards database [6].

To construct a large and robust integrated discovery dataset for RA diagnostic model development and the identification of disease-associated metabolic plasticity patterns, multiple transcriptomic cohorts were harmonized (n = 2,350; Table S3). To remove batch effects across datasets, the ComBat() function from the ‘sva’ R package [7] was applied, while preserving biological variation associated with disease status. Hereafter, this dataset is referred to as the ComBat-integrated dataset. It was used for model training and downstream biological analyses, whereas independent transcriptomic cohorts were reserved for external validation. Samples were stratified into four clinical groups: rheumatoid arthritis (RA), osteoarthritis (OA), systemic lupus erythematosus (SLE), and healthy controls (Figure. 2A-B). After batch correction, principal component analysis (PCA) was performed to confirm that disease-related biological heterogeneity was preserved in the ComBat-integrated datasets (Figure. 2B).

2. Establishment of the directed PMP interaction score

Based on our previous studies [8,9], we developed MMP^{3}C v2 to quantify directed pairwise metabolic plasticity (PMP) interaction between two metabolic pathways by integrating normalized gene expression with protein-protein interaction (PPI) network topology [10]. Using 84 KEGG metabolic pathways, we computed directed PMP interaction scores for all ordered pathway pairs (excluding self-pairs), yielding 6,960

directed comparisons (Table S1-2). For each ordered pathway pair MP_a ➔ MP_b, we computed a per-sample directional score S(a➔b) that aggregates gene-level expression differences between genes in MP_a h and their nearest neighbors in MP_b on the PPI graph, weighing each contribution by the reciprocal of the shortest distance between gene to network. The shortest path calculation was implemented using the closed algorithm in Python (version 3.10.9).

Human protein interactome

The human protein interactome knowledge in this study was constructed from multiple sources of experimentally validated protein interactions. These included: (i) binary interactions from high-throughput yeast two-hybrid experiments, three-dimensional protein structures; (ii) interactions detected by affinity purification coupled by mass spectrometry; (iii) kinase substrate interactions; (iv) signaling interactions; and (v) regulatory interactions. The resulting network comprised 18,505 proteins and 327,924 interactions [10].

Directional pairwise metabolic plasticity (PMP) score — formal definition

Let MPa and MPb denote the gene sets of two metabolic pathways A and B, respectively. For each gene g ∈ MPa, we define the network distance between g and MPb on the PPI as

$$h_g = \arg \min_{h \in MP_b} d(g,h)$$

Where d(u,v) is the shortest-path distance between node u and subnetwork v in the PPI graph. For sample (or cell) s, denote normalized expression by Eg(.). The gene expression matrix was normalized as $\log_2(\mathrm{TPM} + 1)$ to remove the effect of confound factors such as gene length and sequence depth. The per-sample raw directed pairwise metabolic plasticity (PMP) score from MPa to MPb is computed as the sum over genes in MPa of the weighted expression differences between each source gene and its nearest target endpoint. Specifically, for any two metabolic pathway gene sets A and B, the metabolic plasticity score was calculated as follows: for each gene in gene set A, the difference in gene expression values between its corresponding node and the shortest path node in gene set B was divided by the length of the shortest path. These values were then weighted and summed up to derive the final PMP interaction score.

$$PMP\ \mathrm{score}_{a \to b}^{s} = \sum_{s \in MP_a} \frac{E_s(source) - E_s(target)}{d(source,\ b)}$$

To avoid zero-distance artifacts, genes belong to both MPa and MPb are, genes that belong to both MPa and MPb are, for the directed calculation MPa➔MPb, treated as members of the source MPa and excluded from the target set MPb. This assignment is applied directionally (the reverse direction MPb➔MPa treats shared genes as members of MPb). Pathway pairs in which one pathway is entirely contained within the other

were identified and excluded from analysis (see Table S2).

Per-sample normalization for cross-sample comparisons

After computing the directed score $S_{a\to b}^{s}$ for every ordered pathway pair and sample, we assembled each sample's PMP interaction score vector

$$v_s = (S_{1\to 2}^{(s)}, S_{1\to 3}^{(s)}, \dots, S_{83\to 84}^{(s)})$$

To make PMP profiles comparable across samples while preserving relative pairwise patterns, we applied cosine (L2) normalization to each sample vector:

$$\grave{v_s} = \frac{v_s}{\sqrt{\sum_{a\neq b}(S_{a\to b}^{(s)})^2}}$$

The normalized vector was used in all cross-sample analyses such as group comparisons, and diagnosis model training and testing. This step mitigates global magnitude differences across samples while keeping the relative signature of directed pathway interactions.

SHAP analysis unveiling the gene-level contribution of metabolic plasticity program in RA compared with healthy controls

MMP^{3}C v2 incorporates a regression module implemented in the 'glmnet' R package (V4.1.10) [11] to fit a least absolute shrinkage and selection operator (LASSO) regression model, in which adjusted gene-pair difference features were used as predictors and the corresponding PMP score was used as the response variable. This module prioritizes candidate driver genes associated with each PMP score. After model fitting, SHAP analysis using the 'fastshap' R package (V0.1.1) [12] was performed to quantify the gene-level contribution score to the final PMP pattern.

3. Immune-related metabolic plasticity interaction signatures

WGCNA analysis identifying immune-related metabolic plasticity signatures

After gaining the metabolic plasticity matrix, Weighted Gene Co-expression Network Analysis (WGCNA) was performed using 'WGCNA' [13] R package (V1.72.5) to identify immune-related metabolic plasticity modules. Immune-related scores were computed by the Estimation of STromal and Immune cells in MAlignant TIssue using Expression data (ESTIMATE) and Immunophenoscore (IPS) methods using the 'estimate' [14] and 'IOBR' [15] R packages respectively. The correlation between immune-related scores and metabolic plasticity modules was calculated by Pearson

correlation coefficient using the cor() function in R software. To filter meaningful correlations, a cutoff of |0.5| was applied to the Pearson correlation coefficient, retaining only strong positive (⩾ 0.5) or negative (⩽ -0.5) associations.

Integrated analysis for detecting immune-related metabolic plasticity signatures

We employed an integrative framework adapted from the ImmLnc method [16], an integrated framework for identifying long noncoding RNA (lncRNA) modulators of immune-related pathways. Specifically, genes within metabolic plasticity signatures were utilized as seed genes in a PPI network retrieved from STRING database [17]. Then, network propagation was performed using 'networkX' python package [18] to assign the propagation scores to each gene within the PPI network. Finally, the gene set enrichment analysis (GSEA) was conducted via the 'fgsea' V1.34.2 R package [19] to evaluate the associations between metabolic plasticity signatures and 17 immune related pathways obtained from the Immport database [20]. We considered the metabolic plasticity features with the adjusted false discovery rate (FDR) p value <0.05 as significant ones.

4. An RA diagnosis model constructed via the integration of 12 machine learning and deep learning methods

Samples inclusion and exclusion for diagnostic model establishment

For the establishment of RA diagnostic model based on blood transcriptomics, we manually collected the phenotype information of patients from GEO and RABC database and thus selected and excluded the specific samples to ensure the robustness and accuracy of our model. **Inclusion Criteria:** (i) Samples from individuals diagnosed with RA, SLE, OA, and healthy controls were included in the analysis; (ii) Only whole blood and peripheral blood mononuclear cells were considered for inclusion, as these are commonly used and clinically relevant tissue types; (iii) All disease diagnoses (RA, SLE, and OA) were confirmed according to established clinical criteria. **Exclusion Criteria:** (i) Samples from patients undergoing medication at the time of sample collection were excluded from the training dataset (Combat-integrated dataset); (ii) Samples with poor quality or missing key gene expression were excluded to ensure the integrity and consistency of the analysis (Table S3).

Feature selection pipeline

We established a meta-analysis-based feature selection pipeline to obtain robust and reproducible PMP signatures across diverse cohorts and heterogeneous technology platforms. Differential PMP signatures from five case-control cohorts, prior to batch correction in training data, were identified using 'limma' R package (adjusted for age

and sex). The results of these differential PMP signatures were then integrated using 'metafor' V4.8.0 R package [21]. The associations between immune-related metabolic plasticity features and clinical outcomes (RA vs non-RA) were systematically evaluated. Feature selection was performed through a two-step rigorous process: (1) Pearson correlation coefficients between each feature and the outcome variable were calculated, and features with an absolute correlation >0.5 were retained; (2) the area under the curve (AUC) was computed for each remaining feature, and only those with AUC >0.6 were included in the final modeling phase. Next, 44 significant signatures were utilized for RA diagnostic model construction via the integration of 12 machine learning and deep learning methods. In addition, the Combat-integrated dataset was used to train and test RA diagnostic models. The 12 independent validation datasets (Table S3) were then used to evaluate the performance and generalizability of the established models.

The machine and deep learning model construction for RA diagnosis

To establish the RA diagnostic model, 12 machine learning and deep learning methods consisting of LASSO, elastic net, ridge regression, stepwise regression, Gradient Boosting Machine (GBM), extreme gradient boosting (XGBoost), light GBM, Supervised Principal Component Analysis (SuperPC), Partial Least Squares (PLS), support vector machine (SVM), random forest (RF), and Multilayer Perceptron neural network (MLP) were utilized. A unified machine learning integration pipeline was implemented using both R (caret and related machine learning R packages) and Python (scikit-learn and Optuna). Specifically, we first defined an initial feature set comprising 44 candidate PMP features previously identified. Then, diverse methods including step regression (forward, backward, and bidirectional), LASSO, ridge regression, elastic net, RF, XGB, Support Vector Machine-Recursive Feature Elimination (SVM-RFE) were used to filter important features in the ComBat-integrated dataset. Features that were consistently selected across multiple methods (n >= 4) were aggregated into a combined feature set. Subsequently, 11 machine and deep learning algorithms (LASSO, elastic net, ridge regression, GBM, XGBoost, LightGBM, SuperPC, PLS, SVM, RF, MLP) were performed on the 10 selected PMP feature sets. By systematically integrating the feature selection methods with these classification algorithms, 110 distinct hybrid model combinations were constructed and trained on the ComBat-integrated dataset. The optimal hyperparameters for each model were determined by employing 5-fold cross-validation strictly within the training dataset, and the final models were fitted using these settings. Crucially, to prevent any risk of data leakage, these final fitted models were subsequently evaluated on independent validation datasets that were kept completely isolated from the preprocessing, feature selection, and hyperparameter tuning phases.

Initial model benchmarking was conducted using a unified machine learning integration pipeline implemented in both R and Python environments. In the R-based workflow (R V4.5.1), feature selection and model construction were performed using the following packages: LASSO (R package 'glmnet' V4.1.10) [11], stepwise regression (R package 'stats' V4.5.1), GBM (R package 'gbm' V2.2.2) [22], light GBM (R package

‘lightgbm’ V4.6.0) [23], XGBoost (R package ‘XGBoost’, V1.7.5.1) [24], SuperPC (R package ‘superpc’ V1.12), PLS (R package ‘pls’ V2.8.5), RF (R package ‘randomForest’, V4.6-14) [25], SVM (R package ‘e1071’ V1.7-7) [26], and MLP (R package ‘neuralnet’, V3.32.1.3) [27]. In the Python-based workflow (Python V3.10.9), model development relied on the Python packages scikit-learn (V1.2.1) [28], xgboost (V2.0.3) [24], and lightgbm (V3.3.5) [29], together with Optuna (V4.4.0) [30] for hyperparameter tuning. Hyperparameters were optimized independently for each model using Optuna with the Tree-structured Parzen Estimator (TPE) sampler. Model performance was estimated by stratified 5-fold cross-validation, and the mean ROC AUC across folds was used as the optimization objective. To improve efficiency, we restricted the search space to empirically reasonable ranges and applied Optuna’s MedianPruner to stop unpromising trials early. The best hyperparameter set for each model was selected according to the highest cross-validated AUC. After comparison under the same evaluation framework, the Python-based pipeline was selected because it was computationally more efficient and yielded better predictive performance.

Benchmark test between our RA diagnostic model and previously published biomarkers/models

The discriminative performance of our StepRidge model was compared with that of seven previously published models across 12 independent validation datasets (Table S3) using the AUC and F1 score metrics. Initially, the directionality of predicted probabilities output by the diagnostic model was aligned with RA label. Specifically, the ‘direction’ parameter in the auc() function of the ‘pROC’ R package was configured according to the original directionality reported in the literature for each model, ensuring an accurate and fair performance comparison. If the model’s directionality was not reported, the directionality of predicted probabilities from the Combat-integrated dataset was used. All models were implemented according to their original publications, and the corresponding prediction rules, cutoffs, or coefficients were applied as reported (Table S5). The validation datasets were processed using a harmonized preprocessing pipeline before model evaluation. When model-specific preprocessing requirements were described in the original papers, we followed those procedures as closely as possible; otherwise, the same standardized preprocessing workflow was applied across models. We did not retrain the published models on our datasets. Instead, all models were directly tested on the same external cohorts to ensure an unbiased comparison of AUC and F1 score. Optimal performance metrics, including specificity, sensitivity, precision, recall, and cutoff, were determined using Youden index for each validation dataset. Next, F1 score was calculated for each validation dataset based on three predefined decision thresholds: (i) the optimal cutoff point derived from the Youden index; (ii) the cutoff point of training dataset; and (iii) the fixed cutoff point of 0.5.

5. Clinical simulation for screening analysis

We use the Bayesian Posterior Probability Index (BPPI), defined as follows:

$$BPPI = \frac{Sensitivity * Prevalence}{Sensitivity * Prevalence + (1 - Specificity) * (1 - Prevalence)}$$

We applied the BPPI index in a simulated clinical screening scenario to compare RA diagnostic models. For each validation dataset (sample size > 100), we calculated the sensitivity and specificity of the diagnostic models based on the cutoff point derived from the Youden index. These metrics were then averaged across validation datasets. Prevalence data for RA among individuals aged 25 years and above were obtained from the 2023 Global Burden of Disease (GBD) database, covering global data as well as data categorized by the Socio-demographic Index (SDI) regions [31,32].

6. Single-cell RNA-seq analysis

Data collection, preprocess, cell clustering, and annotation

The scRNA-seq datasets were retrieved from public repositories. The CELLxGENE dataset, which includes 18 RA and 18 healthy control samples from peripheral blood, and two scRNA-seq datasets (GSE246416 and GSE200815), comprising 4 RA and 5 healthy control samples from synovial tissue, were downloaded from the GEO database. Additionally, the ArrayExpress dataset E-MTAB-8322 was downloaded from ArrayExpress database [33]. Sample accession numbers along with detailed metadata are provided in Table S11.

We performed quality control, cell clustering and cell annotation for each scRNA-seq dataset. The resulting cell subpopulations from different datasets were then integrated using ‘Harmony’ method for further cell clustering and annotation [34]. We preprocessed single-cell gene expression data utilizing the ‘Seurat’ [35] R package (V 5.1.0). For peripheral blood scRNA-seq dataset, the VlnPlot() function was employed to visualize quality control indices and filter low-quality cells with fewer than 200 UMIs, gene count below 200, mitochondrial gene percentages exceeding 20%, ribosomal gene percentages exceeding 66%, hemoglobin gene number exceeding 3, and gene-to-UMI ratio below 0.8. For synovial scRNA-seq dataset, low-quality cells with fewer than 200 UMIs, gene count below 200, mitochondrial gene percentages exceeding 25%, ribosomal gene percentage exceeding 60%, hemoglobin gene number exceeding 3, and gene-to-UMI ratio below 0.8 were filtered. Cell subpopulations exhibiting distinct cell type-specific features were identified as doublets and subsequently removed from the analysis [34].

After the quality control process, the gene expression matrix underwent library size normalization via NormalizeData() function with “LogNormalize” method and a size factor of 1,000. Highly variable genes were identified using the "vst" method in FindVariableFeatures(), and principal component analysis (PCA) was performed with the top 2,000. Furthermore, the number of highly variable features was set to 1,500 for clustering of single-cell subtypes (e.g., T cells, fibroblasts, and myeloid cells). The optimal number of PCs was determined by PCElbowPlot(). Cell clustering was

executed with FindNeighbors() and FindClusters(), followed by dimensionality reduction using RunUMAP() for visualization. Differentially expressed genes (DEGs) were identified within each cluster using the Wilcoxon rank-sum test in FindAllMarkers() or FindMarkers(), with an adjusted p-value thresh-old of 0.05. These DEGs were then used to annotate cell subtypes, referencing the CellMarker V2.0 database [36] and prior research. Cell subpopulations exhibiting distinct cell type-specific features were identified as doublets and subsequently removed from the analysis [34]. The IntegrateLayers() function in 'Seurat' R package was utilized to integrate multiple single-cell transcriptome data charts. The method parameter 'harmony' was used in the data integration process. Additionally, we used Seurat's SelectIntegrationFeatures() function to select the highly consistent variable genes across samples, which were subsequently used for downstream integration.

Cross-tissue Trajectory Analysis to infer T cell fates in the RA process

The T cells comprising RA and healthy control samples from synovial and blood tissue were further performed T cell clustering and annotation. Then, the trajectory inference analysis using the 'Monocle2' [37] R package (V2.3.6) revealed the different T cell fates in the RA process. DEGs among T cell subclusters identified by Seurat were regarded as ordering genes. We established the root state as naïve T cells and applied the orderCell() function from Monocle2. Additionally, to account for unequal cell numbers across tissues and disease states, we downsampled T cells using Seurat's sketching method [35], and repeated this process to confirm robustness (Figure S7).

Inferring the association of PMP patterns with RA in each cell

Per-cell disease relevance was quantified using the scDRS framework (Single-cell Disease Relevance Score; v1.0.3) [38]. Two independent GWAS datasets (GCST90044540 and finngen_R10_M13_RHEUMA) from the GWAS Catalog and FinnGen consortium were analyzed, respectively. For each GWAS, we first performed gene-level association analysis using MAGMA (v1.10) [39] based on the corresponding summary statistics and gene annotation files from the 1000 Genomes European reference panel. The top 1,000 genes ranked by MAGMA gene-level p values were defined as the disease-associated gene set. For each cell, scDRS computed an aggregated expression score of the disease gene set and compared it with 1,000 matched control gene sets selected by mean expression and expression variance. Default parameters were used (n_ctrl = 1000, matching = "mean_var"), and per-cell empirical p-values were obtained from the null distribution. The resulting disease scores were z-normalized across all cells, and Benjamini–Hochberg correction was applied to control the false discovery rate (FDR < 0.05).

Cell-cell communication analysis

We used the 'CellChat' [40] R package (V1.6.1) to perform cell-cell communication analysis in synovial tissue between RA patients and healthy controls. A CellChat object was created by normalized gene expression single-cell matrix derived from Seurat object, using the human ligand-receptor interaction database (CellChatDB.human).

Overexpressed ligands and receptors in each cell type were identified with identifyOverExpressedGenes() function and identifyOverExpressedInteractions() function. Communication probabilities between cell types were calculated using computeCommunProb() function and low-probability interactions were filtered with filterCommunication(). Key signaling pathways were identified to highlight significant sender-receiver interactions across cell types or disease states (RA vs healthy controls).

Imputation of single-cell expression matrix by DeepImpute

To mitigate the influence of dropout events in single-cell RNA-seq data, we performed expression imputation using DeepImpute (V1.2) [41]. In this study, we applied imputation only to metabolic genes, defined as those annotated in the KEGG metabolic pathways (n = 84 pathways). Non-metabolic genes were retained in their original normalized form to avoid introducing potential bias into non-metabolic transcriptional programs. We used the "genes_to_impute" argument in MultiNet.fit() to explicitly specify the metabolic gene set as the target gene to be imputed. DeepImpute was then trained on the raw count expression matrix with default parameters, and the imputed values corresponding to metabolic genes were extracted and merged back into the full expression matrix for downstream analyses.

Virtual knockout of FN1 in RA-derived Myofibroblasts using scTenifoldKnk

To validate the potential association between FN1 and metabolic pathways, a virtual knockout of FN1 was performed in RA-derived myofibroblasts using the Python package 'scTenifoldKnk' (V0.1.3) [42] with default parameters. Subsequently, the resulting Z-scores of the perturbed genes were subjected to GSEA using the R package 'clusterProfiler' (V4.16.0) [43] under default settings to specifically evaluate the enrichment of metabolic pathways from KEGG database.

7. Statistical analysis

Statistical analyses and data visualizations were primarily performed using R software (version 4.5.1) and Python (version 3.9.10). For the comparison of continuous variables, the Student's t-test was employed for normally distributed data, whereas the Wilcoxon rank-sum test was utilized for non-normally distributed data. Correlation analyses were conducted using Pearson or Spearman correlation coefficients as appropriate. Multiple testing correction was performed using the FDR approach. ROC curves were used to estimate the classification performance via R package 'pROC' (V1.18.0) [44]. Data visualizations were mainly constructed utilizing the 'ggplot2' R package [45]. All statistical tests were two-sided, and a P-value < 0.05 (or FDR < 0.05 where applicable) was considered statistically significant.

Supplementary Figures

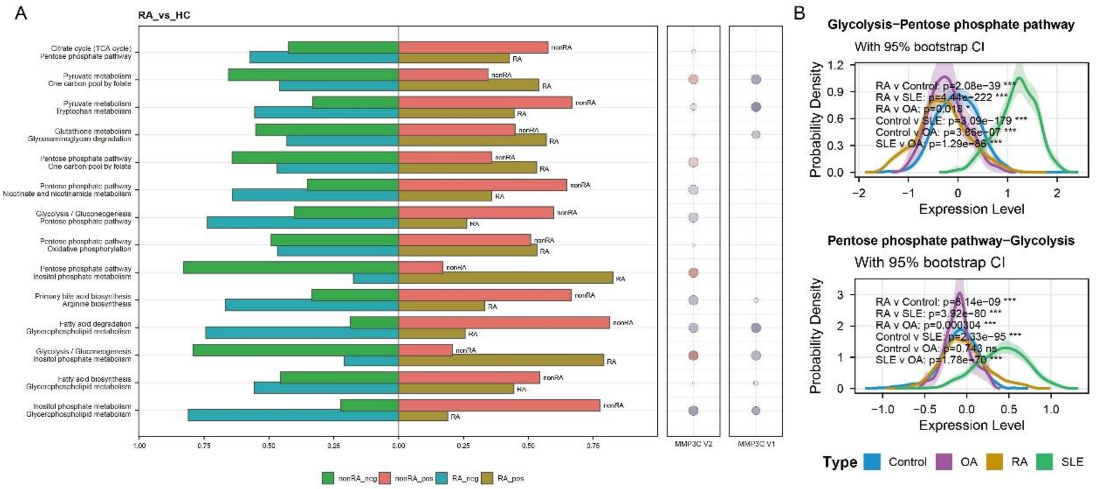

Figure S1 (A) Detecting performance comparison of MMP^{3}C v2 and MMP^{3}C v1 on Combat-integrated bulk transcriptomic datasets. (B) The density distribution plots of bidirectional normalized PMP interactions across various disease states including RA, healthy controls, SLE, and OA. Shown are representative pathway pair: Glycolysis → Pentose phosphate pathway. PMP, pairwise metabolic pathway; OA, osteoarthritis; SLE, systemic lupus erythematosus.

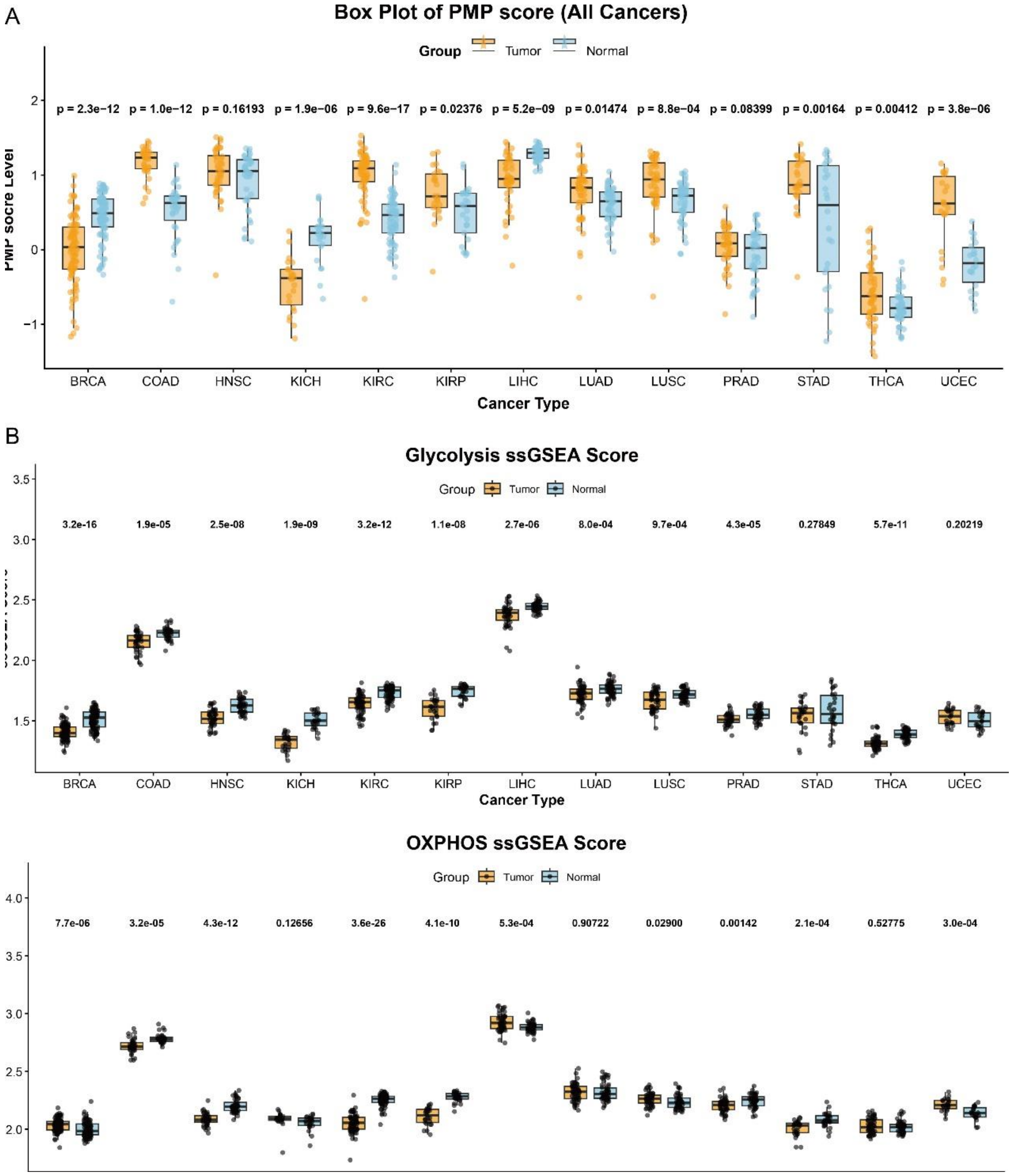

Figure S2 (A) Detecting performance of MMP^{3}C v2 for Warburg effect between glycolysis and oxidative phosphorylation on pan-cancer bulk transcriptomic datasets from TCGA database. (B) The bar plots of glycolysis (up) and OXPHOS (down) enrichment score computed by ssGSEA method. OXPHOS, oxidative phosphorylation; ssGSEA, single sample gene set enrichment analysis.

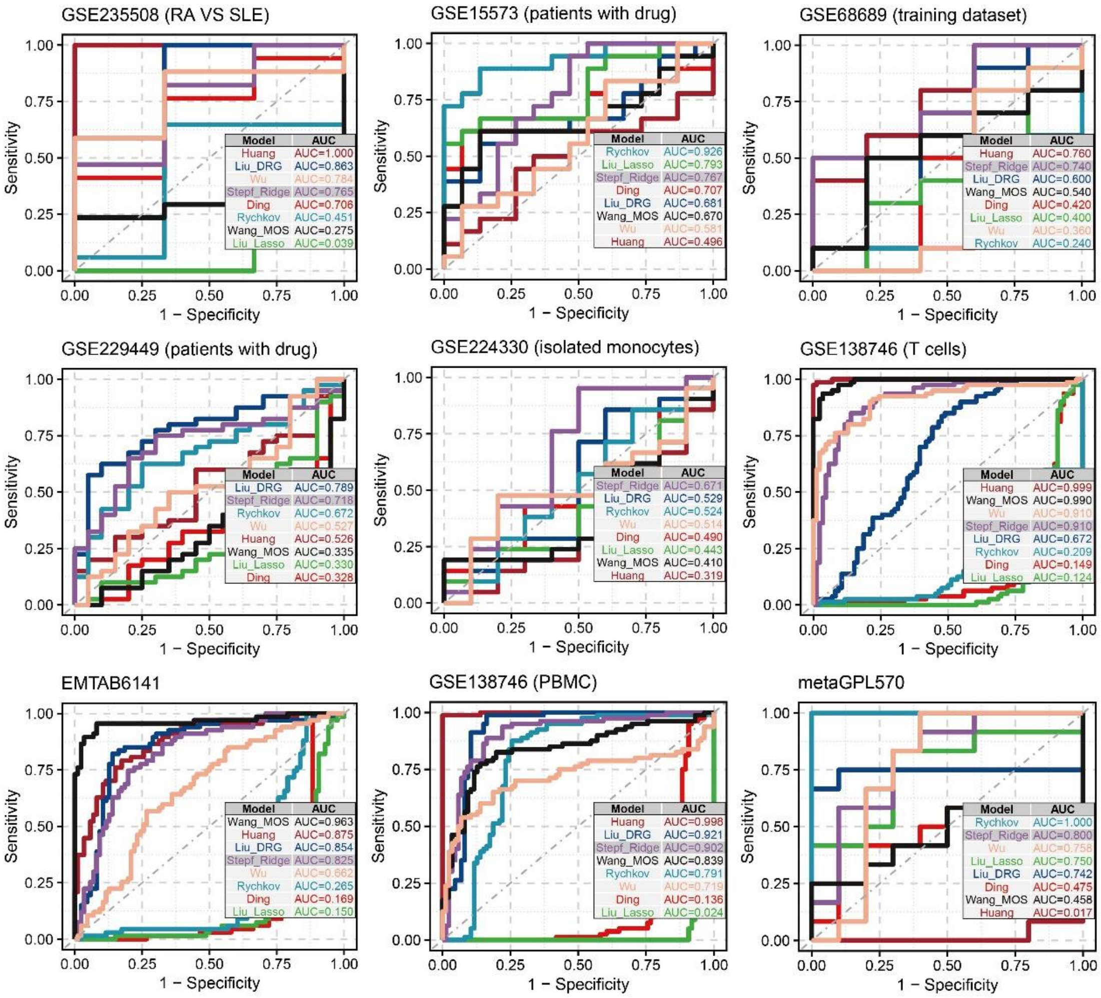

Figure S3 ROC curves comparing the StepfRidge models with seven previously published models on the training and validation datasets.

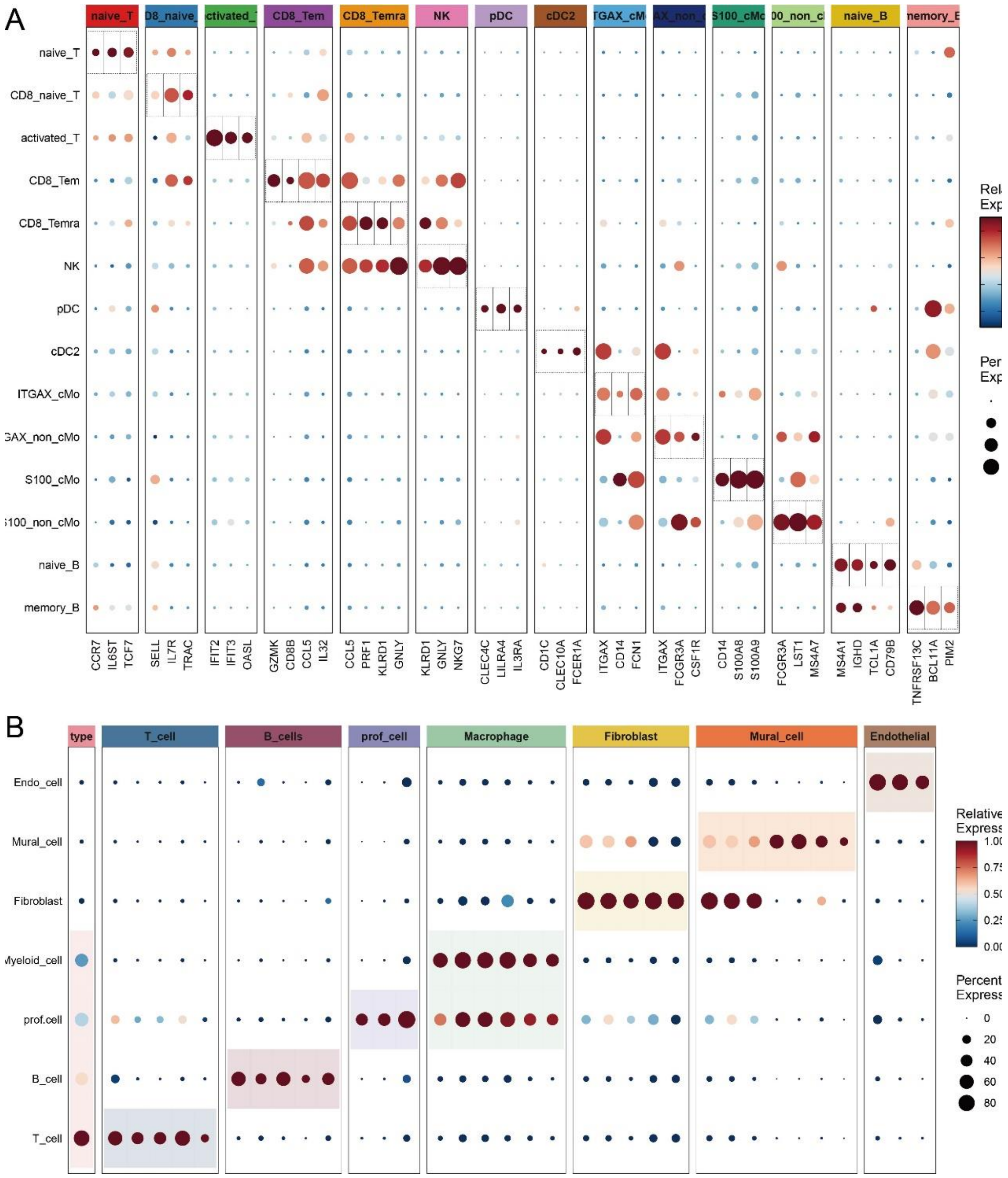

Figure S4 (A) Dot plot showing expression of representative marker genes across cell types in peripheral blood single-cell RNA-seq data (CELLxGENE database). (B) Dot plot showing expression of representative marker genes across cell types in synovium single-cell RNA-seq dataset (E-MTAB-8322).

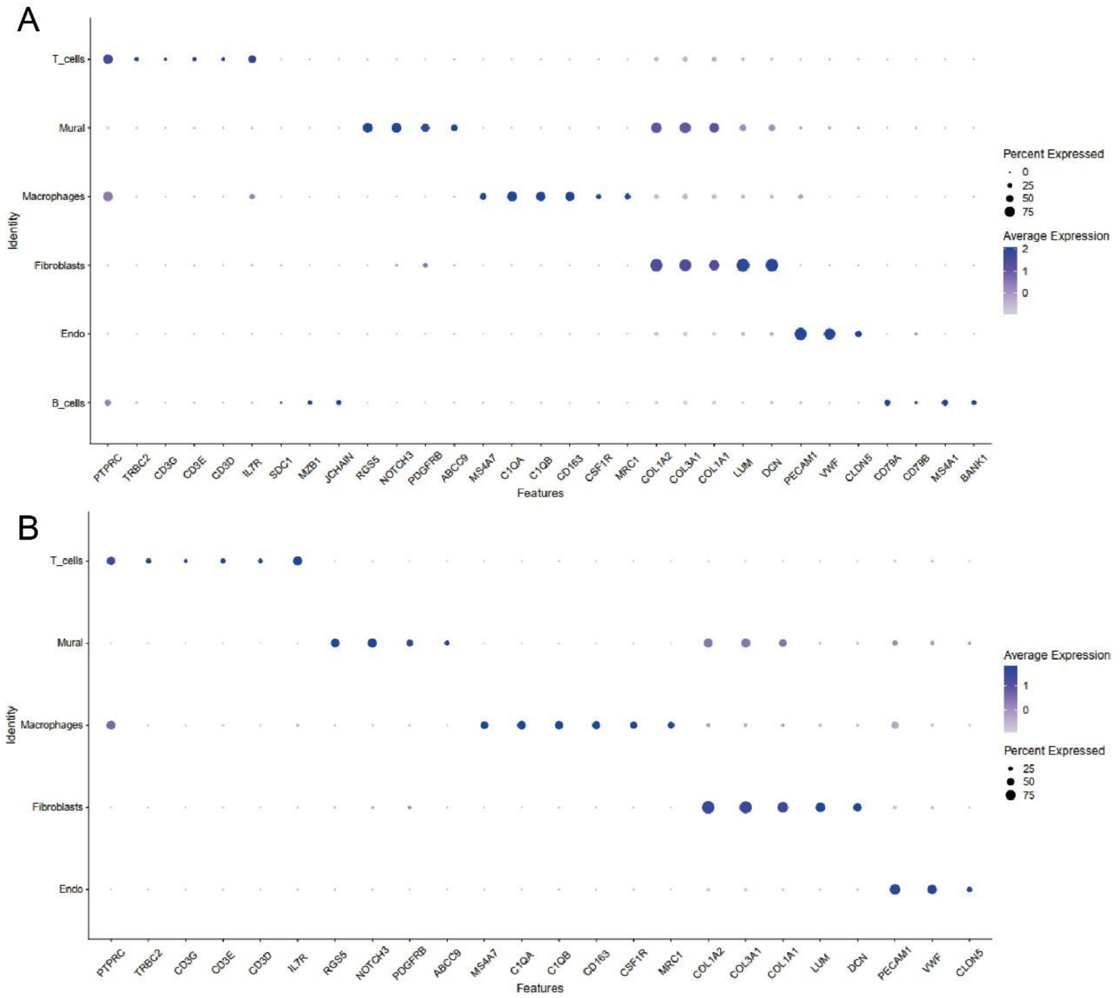

Figure S5 Dot plot showing expression of representative marker genes across cell types in synovium single-cell RNA-seq dataset including (A) GSE246416 and (B) GSE200815.

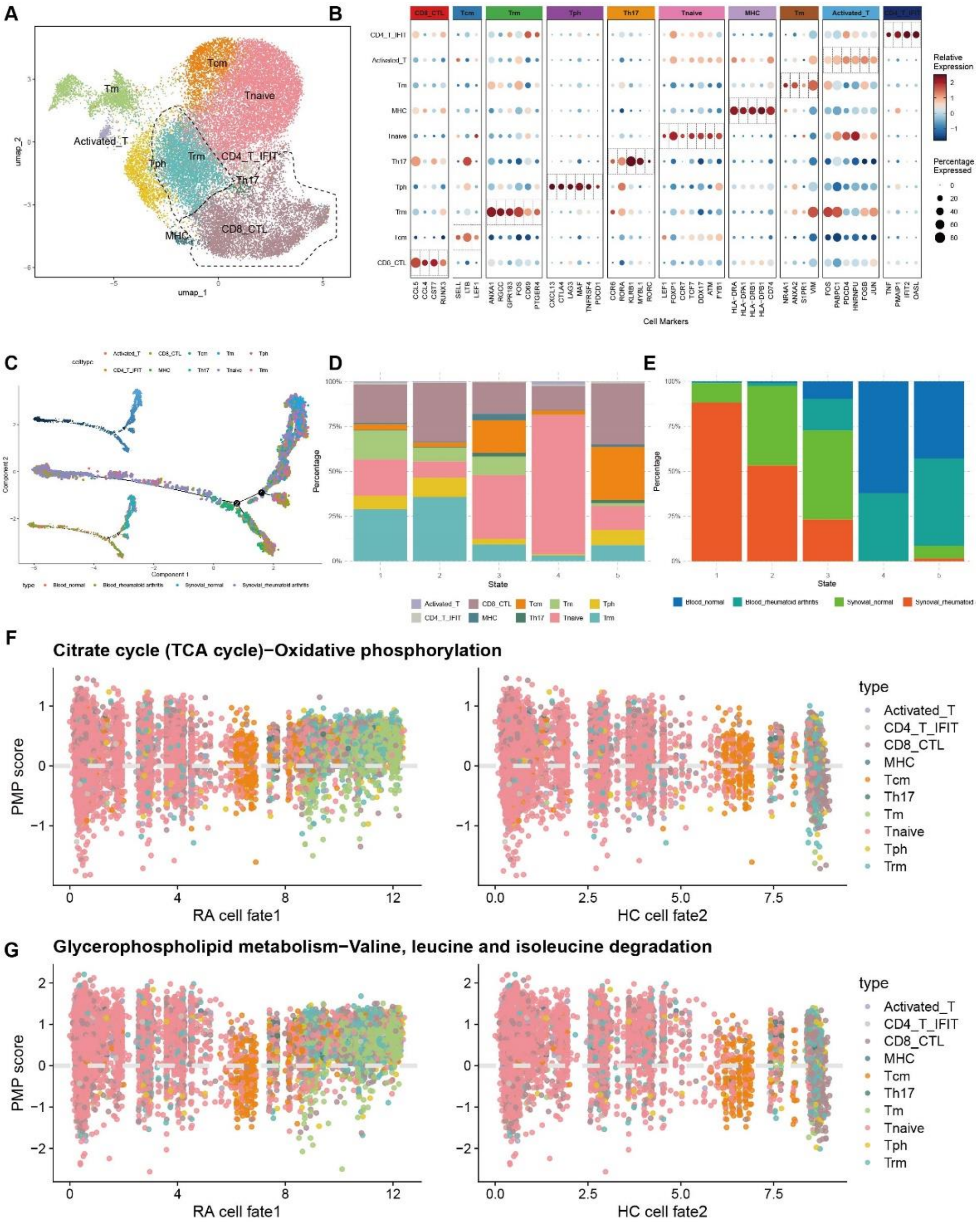

Figure S6 The identification of fate-associated PMP interaction patterns by cross-tissue T cells analysis and trajectory analysis. (A) UMAP plot of 24,143 T cells in RA patients and health controls from peripheral blood and synovium tissues. (B) Dot plot of significant markers of each T cell subtype. (C) Cross-tissue potential trajectory of all T cells identified distinct cell fates. (D) Percent bar plot reveals the distribution of T cell subtypes in various trajectory states. (E) Percent bar plot reveals the distribution of T cells from different disease status and tissue types in various trajectory states. (F) Dot plots of dynamic activity difference of PMP interaction patterns (TCA cycle and OXPHOS) along two cell fates. (G) Dot plots of dynamic activity difference of PMP interaction patterns (Glycerophospholipid and Valine, leucine and isoleucine degradation) along two cell fates.

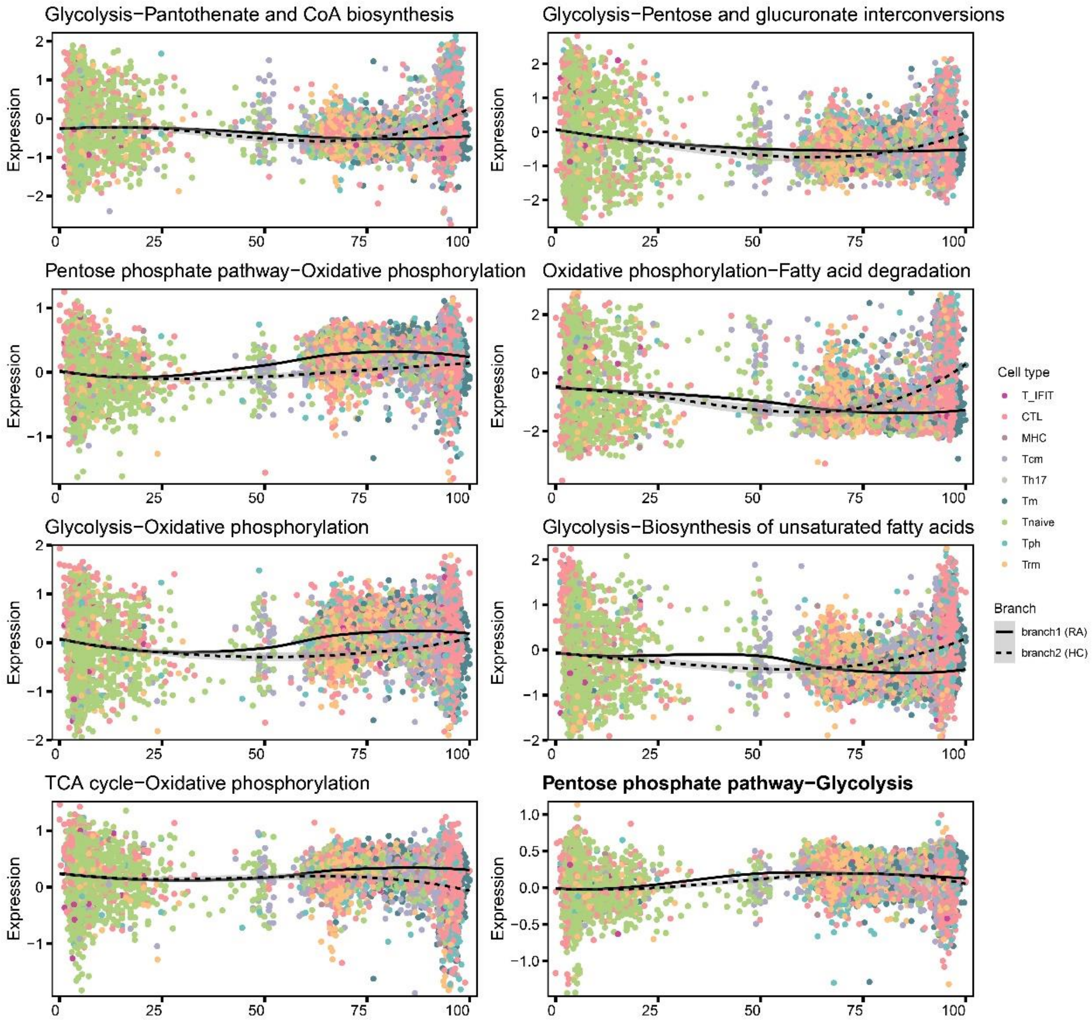

Figure S7 Comparative analysis revealed differential PMP patterns along with T cell trajectory between RA and HC. RA, Rheumatoid Arthritis; HC, Health Control.

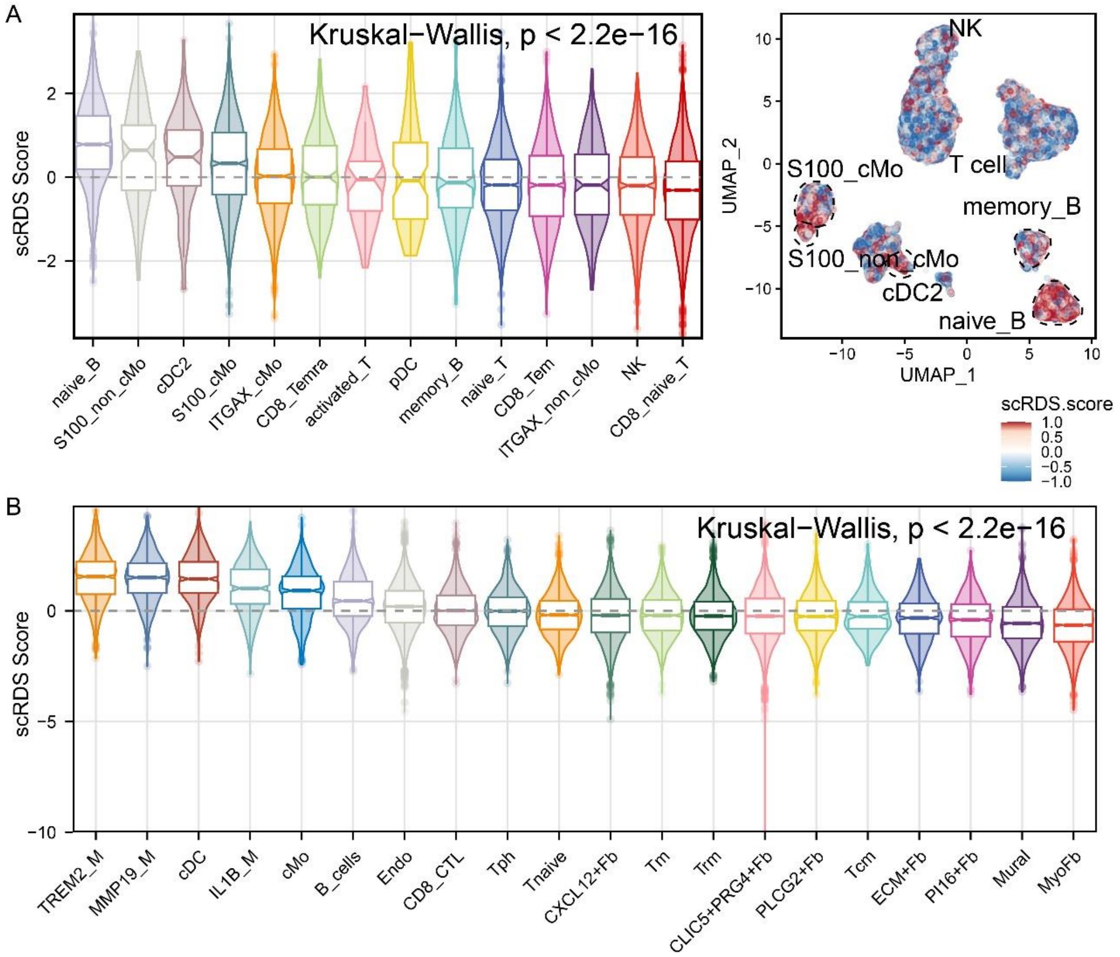

Figure S8 Violin plots display disease risk scores across various cell types in (A) peripheral blood and (B) synovial microenvironment, based on the GWAS dataset (GCST90044540).

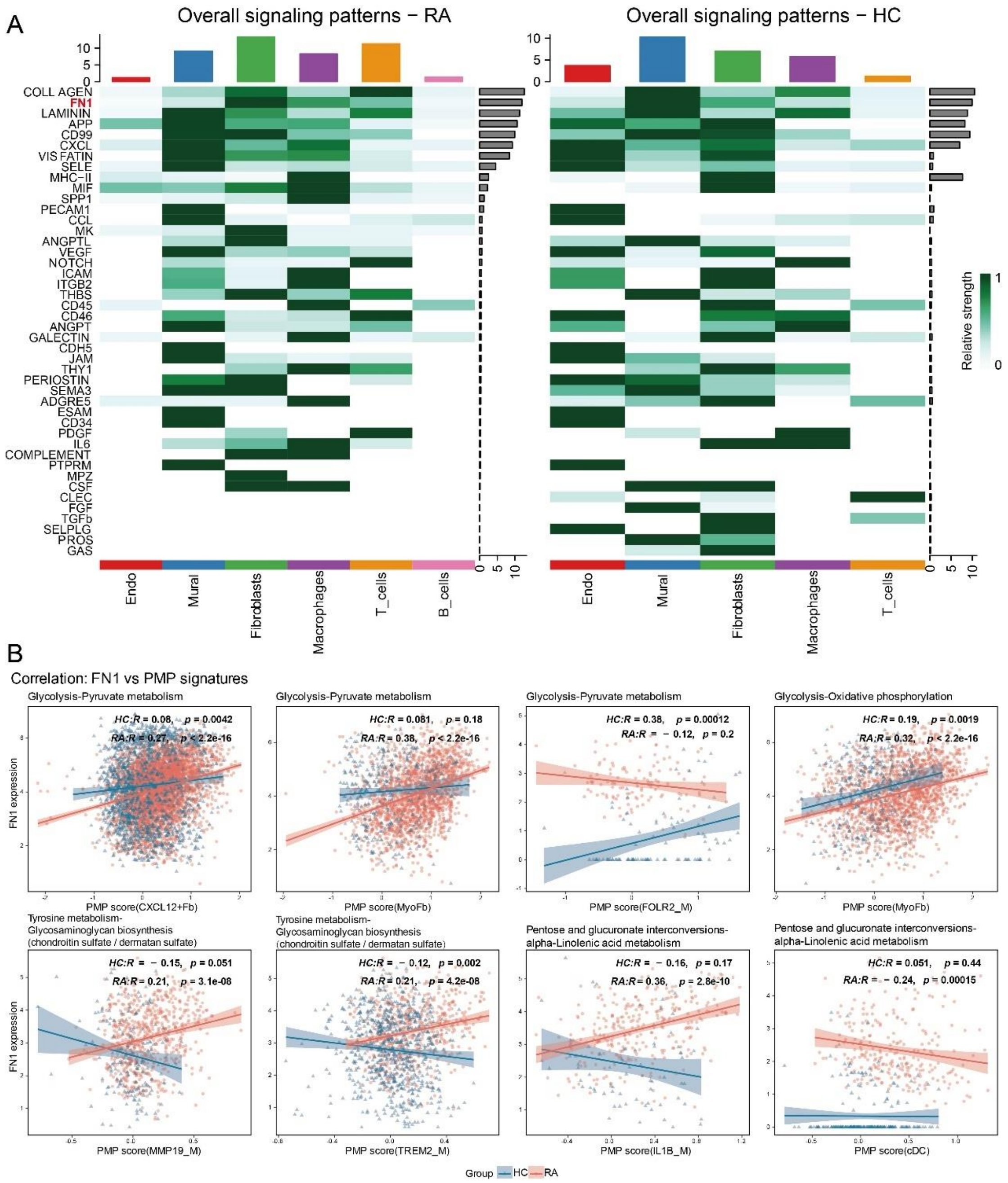

Figure S9 (A) Heatmap illustrates differential signaling patterns between RA and Healthy Controls. (B) Correlation analysis illustrates FN1-associated PMP interaction patterns across multiple cell types in the RA immune microenvironment. PMP, pairwise metabolic pathway; RA, Rheumatoid Arthritis; HC, Health Control.

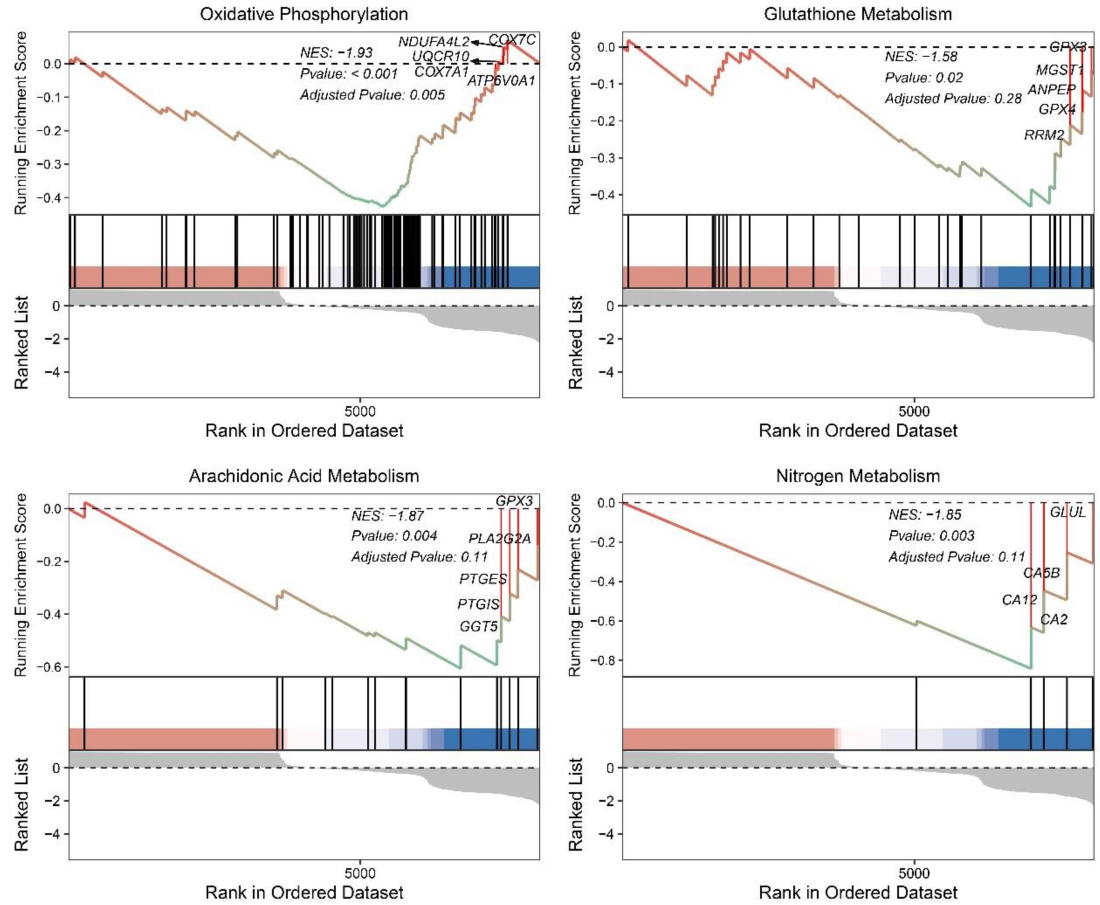

Figure S10 GSEA of metabolic pathways based on Z-scores of perturbed genes following the virtual knockout of *FN1* in RA-derived myofibroblasts.

Reference

1. Edgar, R.; Domrachev, M.; Lash, A.E. Gene Expression Omnibus: NCBI gene expression and hybridization array data repository. *Nucleic Acids Res* **2002**, *30*, 207-210, doi:10.1093/nar/30.1.207.
2. Chen, H.; Xu, J.; Wei, S.; Jia, Z.; Sun, C.; Kang, J.; Guo, X.; Zhang, N.; Tao, J.; Dong, Y.; et al. RABC: Rheumatoid Arthritis Bioinformatics Center. *Nucleic Acids Res* **2023**, *51*, D1381-D1387, doi:10.1093/nar/gkac850.
3. Gautier, L.; Cope, L.; Bolstad, B.M.; Irizarry, R.A. affy--analysis of Affymetrix GeneChip data at the probe level. *Bioinformatics* **2004**, *20*, 307-315, doi:10.1093/bioinformatics/btg405.
4. Ritchie, M.E.; Phipson, B.; Wu, D.; Hu, Y.; Law, C.W.; Shi, W.; Smyth, G.K. limma powers differential expression analyses for RNA-sequencing and microarray studies. *Nucleic Acids Res* **2015**, *43*, e47, doi:10.1093/nar/gkv007.
5. Wu, C.; Macleod, I.; Su, A.I. BioGPS and MyGene.info: organizing online, gene-centric information. *Nucleic Acids Res* **2013**, *41*, D561-565, doi:10.1093/nar/gks1114.
6. Stelzer, G.; Rosen, N.; Plaschkes, I.; Zimmerman, S.; Twik, M.; Fishilevich, S.; Stein, T.I.; Nudel, R.; Lieder, I.; Mazor, Y.; et al. The GeneCards Suite: From Gene Data Mining to Disease Genome Sequence Analyses. *Curr Protoc Bioinformatics* **2016**, *54*, 1 30 31-31 30 33, doi:10.1002/cpbi.5.
7. Leek, J.T.; Johnson, W.E.; Parker, H.S.; Jaffe, A.E.; Storey, J.D. The sva package for removing batch effects and other unwanted variation in high-throughput experiments. *Bioinformatics* **2012**, *28*, 882-883, doi:10.1093/bioinformatics/bts034.
8. Chen, X.; Deng, M.; Wang, Z.; Huang, C. MMP3C: an in-silico framework to depict cancer metabolic plasticity using gene expression profiles. *Brief Bioinform* **2023**, *25*, doi:10.1093/bib/bbad471.
9. Feng, Y.; Chen, X.; Zhang, X.D.; Huang, C. Metabolic Pathway Pairwise-Based Signature as a Potential Non-Invasive Diagnostic Marker in Alzheimer's Disease Patients. *Genes (Basel)* **2023**, *14*, doi:10.3390/genes14061285.
10. Gan, X.; Shu, Z.; Wang, X.; Yan, D.; Li, J.; Ofaim, S.; Albert, R.; Li, X.; Liu, B.; Zhou, X.; et al. Network medicine framework reveals generic herb-symptom effectiveness of traditional Chinese medicine. *Sci Adv* **2023**, *9*, eadh0215, doi:10.1126/sciadv.adh0215.
11. Friedman, J.H.; Hastie, T.; Tibshirani, R. Regularization paths for generalized linear models via coordinate descent. *Journal of statistical software* **2010**, *33*, 1-22.
12. Greenwell, B. fastshap: fast approximate Shapley values. *CRAN: Contributed Packages* **2019**.
13. Langfelder, P.; Horvath, S. WGCNA: an R package for weighted correlation network analysis. *BMC Bioinformatics* **2008**, *9*, 559, doi:10.1186/1471-2105-9-559.
14. Yoshihara, K.; Shahmoradgoli, M.; Martinez, E.; Vegesna, R.; Kim, H.; Torres-Garcia, W.; Trevino, V.; Shen, H.; Laird, P.W.; Levine, D.A.; et al. Inferring tumour purity and stromal and immune cell admixture from expression data. *Nat Commun* **2013**, *4*, 2612, doi:10.1038/ncomms3612.
15. Zeng, D.; Ye, Z.; Shen, R.; Yu, G.; Wu, J.; Xiong, Y.; Zhou, R.; Qiu, W.; Huang, N.; Sun, L.; et al. IOBR: Multi-Omics Immuno-Oncology Biological Research to Decode Tumor Microenvironment and Signatures. *Front Immunol* **2021**, *12*, 687975,

doi:10.3389/fimmu.2021.687975.
16. Li, Y.; Jiang, T.; Zhou, W.; Li, J.; Li, X.; Wang, Q.; Jin, X.; Yin, J.; Chen, L.; Zhang, Y.; et al. Pan-cancer characterization of immune-related lncRNAs identifies potential oncogenic biomarkers. *Nat Commun* **2020**, *11*, 1000, doi:10.1038/s41467-020-14802-2.
17. Szklarczyk, D.; Kirsch, R.; Koutrouli, M.; Nastou, K.; Mehryary, F.; Hachilif, R.; Gable, A.L.; Fang, T.; Doncheva, N.T.; Pyysalo, S.; et al. The STRING database in 2023: protein-protein association networks and functional enrichment analyses for any sequenced genome of interest. *Nucleic Acids Res* **2023**, *51*, D638-D646, doi:10.1093/nar/gkac1000.
18. Hagberg, A.; Swart, P.J.; Schult, D.A. *Exploring network structure, dynamics, and function using NetworkX*; Los Alamos National Laboratory (LANL), Los Alamos, NM (United States): 2008.
19. Korotkevich, G.; Sukhov, V.; Budin, N.; Shpak, B.; Artyomov, M.N.; Sergushichev, A. Fast gene set enrichment analysis. *biorxiv* **2016**, 060012.
20. Bhattacharya, S.; Andorf, S.; Gomes, L.; Dunn, P.; Schaefer, H.; Pontius, J.; Berger, P.; Desborough, V.; Smith, T.; Campbell, J.; et al. ImmPort: disseminating data to the public for the future of immunology. *Immunol Res* **2014**, *58*, 234-239, doi:10.1007/s12026-014-8516-1.
21. Viechtbauer, W. Conducting meta-analyses in R with the metafor package. *Journal of statistical software* **2010**, *36*, 1-48.
22. Friedman, J.H. Greedy function approximation: a gradient boosting machine. *Annals of statistics* **2001**, 1189-1232.
23. Shi, Y.; Ke, G.; Soukhavong, D.; Lamb, J.; Meng, Q.; Finley, T.; Wang, T.; Chen, W.; Ma, W.; Ye, Q. lightgbm: light gradient boosting machine. R package version 3.3. 4. **2022**.
24. Chen, T.; Guestrin, C. Xgboost: A scalable tree boosting system. In Proceedings of the Proceedings of the 22nd acm sigkdd international conference on knowledge discovery and data mining, 2016; pp. 785-794.
25. Liaw, A.; Wiener, M. Classification and regression by randomForest. *R news* **2002**, *2*, 18-22.
26. Meyer, D.; Dimitriadou, E.; Hornik, K.; Weingessel, A.; Leisch, F. Misc Functions of the Department of Statistics, Probability Theory Group (Formerly: E1071), TU Wien [R package e1071 version 1.7-4]. 2020.
27. Günther, F.; Fritsch, S. Neuralnet: training of neural networks. *R J.* **2010**, *2*, 30.
28. Pedregosa, F.; Varoquaux, G.; Gramfort, A.; Michel, V.; Thirion, B.; Grisel, O.; Blondel, M.; Prettenhofer, P.; Weiss, R.; Dubourg, V. Scikit-learn: Machine learning in Python. *the Journal of machine Learning research* **2011**, *12*, 2825-2830.
29. Ke, G.; Meng, Q.; Finley, T.; Wang, T.; Chen, W.; Ma, W.; Ye, Q.; Liu, T.-Y. Lightgbm: A highly efficient gradient boosting decision tree. *Advances in neural information processing systems* **2017**, *30*.
30. Akiba, T.; Sano, S.; Yanase, T.; Ohta, T.; Koyama, M. Optuna: A next-generation hyperparameter optimization framework. In Proceedings of the Proceedings of the 25th ACM SIGKDD international conference on knowledge discovery & data mining, 2019; pp. 2623-2631.
31. Ma, Y.; Chen, H.; Lv, W.; Wei, S.; Zou, Y.; Li, R.; Wang, J.; She, W.; Yuan, L.; Tao, J.; et al. Global, regional and national burden of rheumatoid arthritis from 1990 to 2021, with

projections of incidence to 2050: a systematic and comprehensive analysis of the Global Burden of Disease study 2021. *Biomark Res* **2025**, *13*, 47, doi:10.1186/s40364-025-00760-8.

32. Diseases, G.B.D.; Injuries, C. Global incidence, prevalence, years lived with disability (YLDs), disability-adjusted life-years (DALYs), and healthy life expectancy (HALE) for 371 diseases and injuries in 204 countries and territories and 811 subnational locations, 1990-2021: a systematic analysis for the Global Burden of Disease Study 2021. *Lancet* **2024**, *403*, 2133-2161, doi:10.1016/S0140-6736(24)00757-8.

33. Athar, A.; Füllgrabe, A.; George, N.; Iqbal, H.; Huerta, L.; Ali, A.; Snow, C.; Fonseca, N.A.; Petryszak, R.; Papatheodorou, I. ArrayExpress update–from bulk to single-cell expression data. *Nucleic acids research* **2019**, *47*, D711-D715.

34. Yang, Y.; Chen, X.; Pan, J.; Ning, H.; Zhang, Y.; Bo, Y.; Ren, X.; Li, J.; Qin, S.; Wang, D.; et al. Pan-cancer single-cell dissection reveals phenotypically distinct B cell subtypes. *Cell* **2024**, *187*, 4790-4811 e4722, doi:10.1016/j.cell.2024.06.038.

35. Satija, R.; Farrell, J.A.; Gennert, D.; Schier, A.F.; Regev, A. Spatial reconstruction of single-cell gene expression data. *Nat Biotechnol* **2015**, *33*, 495-502, doi:10.1038/nbt.3192.

36. Hu, C.; Li, T.; Xu, Y.; Zhang, X.; Li, F.; Bai, J.; Chen, J.; Jiang, W.; Yang, K.; Ou, Q.; et al. CellMarker 2.0: an updated database of manually curated cell markers in human/mouse and web tools based on scRNA-seq data. *Nucleic Acids Res* **2023**, *51*, D870-D876, doi:10.1093/nar/gkac947.

37. Qiu, X.; Hill, A.; Packer, J.; Lin, D.; Ma, Y.A.; Trapnell, C. Single-cell mRNA quantification and differential analysis with Census. *Nat Methods* **2017**, *14*, 309-315, doi:10.1038/nmeth.4150.

38. Zhang, M.J.; Hou, K.; Dey, K.K.; Sakaue, S.; Jagadeesh, K.A.; Weinand, K.; Taychameekiatchai, A.; Rao, P.; Pisco, A.O.; Zou, J.; et al. Polygenic enrichment distinguishes disease associations of individual cells in single-cell RNA-seq data. *Nat Genet* **2022**, *54*, 1572-1580, doi:10.1038/s41588-022-01167-z.

39. de Leeuw, C.A.; Neale, B.M.; Heskes, T.; Posthuma, D. The statistical properties of gene-set analysis. *Nat Rev Genet* **2016**, *17*, 353-364, doi:10.1038/nrg.2016.29.

40. Jin, S.; Guerrero-Juarez, C.F.; Zhang, L.; Chang, I.; Ramos, R.; Kuan, C.H.; Myung, P.; Plikus, M.V.; Nie, Q. Inference and analysis of cell-cell communication using CellChat. *Nat Commun* **2021**, *12*, 1088, doi:10.1038/s41467-021-21246-9.

41. Arisdakessian, C.; Poirion, O.; Yunits, B.; Zhu, X.; Garmire, L.X. DeepImpute: an accurate, fast, and scalable deep neural network method to impute single-cell RNA-seq data. *Genome Biol* **2019**, *20*, 211, doi:10.1186/s13059-019-1837-6.

42. Osorio, D.; Zhong, Y.; Li, G.; Xu, Q.; Yang, Y.; Tian, Y.; Chapkin, R.S.; Huang, J.Z.; Cai, J.J. scTenifoldKnk: An efficient virtual knockout tool for gene function predictions via single-cell gene regulatory network perturbation. *Patterns (N Y)* **2022**, *3*, 100434, doi:10.1016/j.patter.2022.100434.

43. Xu, S.; Hu, E.; Cai, Y.; Xie, Z.; Luo, X.; Zhan, L.; Tang, W.; Wang, Q.; Liu, B.; Wang, R.; et al. Using clusterProfiler to characterize multiomics data. *Nat Protoc* **2024**, *19*, 3292-3320, doi:10.1038/s41596-024-01020-z.

44. Robin, X.; Turck, N.; Hainard, A.; Tiberti, N.; Lisacek, F.; Sanchez, J.C.; Muller, M. pROC: an open-source package for R and S+ to analyze and compare ROC curves. *BMC*

Bioinformatics **2011**, *12*, 77, doi:10.1186/1471-2105-12-77.
45. Ginestet, C. ggplot2: Elegant Graphics for Data Analysis. *J Roy Stat Soc A* **2011**, *174*, 245-245, doi:DOI 10.1111/j.1467-985X.2010.00676_9.x.
